# Supplementary material for: Spatial arrangement of several flagellins within bacterial flagella improves motility in different environments
Source: Nat Commun. 2018 Dec 18;9:5369. doi: 10.1038/s41467-018-07802-w (PMC6299084; doi:10.1038/s41467-018-07802-w)
Supplement: Supplementary file 1 — Supplementary information [file 41467_2018_7802_MOESM1_ESM.pdf]

Spatial arrangement of several flagellins within bacterial flagella improves motility in different environments

Kühn et al

- Supplementary Information -

This document contains:

Supplementary Figures 1 - 13

Supplementary Tables 1 - 6

Supplementary References

Supplementary Figures

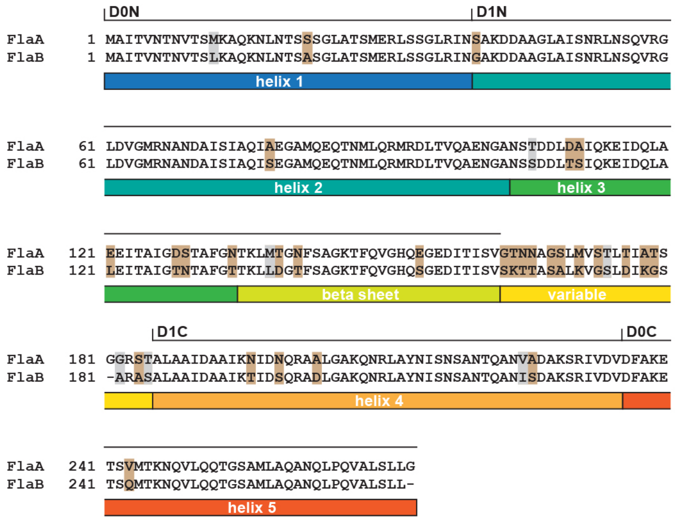

Supplementary Figure 1: Sequence alignment of the polar flagellins of *Shewanella putrefaciens* CN-32. Different residues are marked by brown boxes, different but similar residues are marked by grey boxes. The predicted flagellin domains are given below the sequence. Most differences are located in the variable region, which presumably is exposed to the surrounding medium. The cysteine substitutions sites are also located within this region.

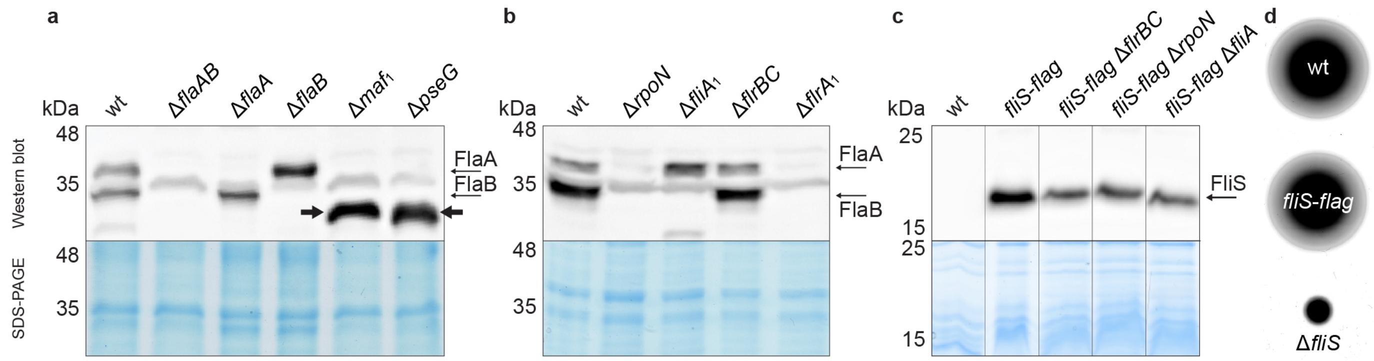

Supplementary Figure 2: Immunoblotting analysis of the polar flagellins and their chaperone FliS. Upper panels = immunoblots, lower panels = corresponding Coomassie-stained PAGE loading controls. **a** Detection of flagellins FlaA and FlaB in flagellin deletion strains and strains lacking two major glycosylation proteins, Maf1 and PseG. The thick arrows indicate the collapsed FlaA and FlaB bands. Likely due to different extents of glycosyl modifications, FlaA and FlaB can be discriminated by their molecular mass (thin arrows). **b** Detection of FlaA and FlaB in strains lacking the sigma factors 54 and 28 (RpoN and FliA, respectively) and other regulatory proteins for flagellar assembly (FlrA and FlrBC). **c** Detection of FLAG-tagged FliS in the same background strains as in **b**. Full blots are shown in Supplementary Fig. 13. **d** Radial expansion of cells producing FLAG-tagged FliS in 0.2% soft agar as control for functional integrity.

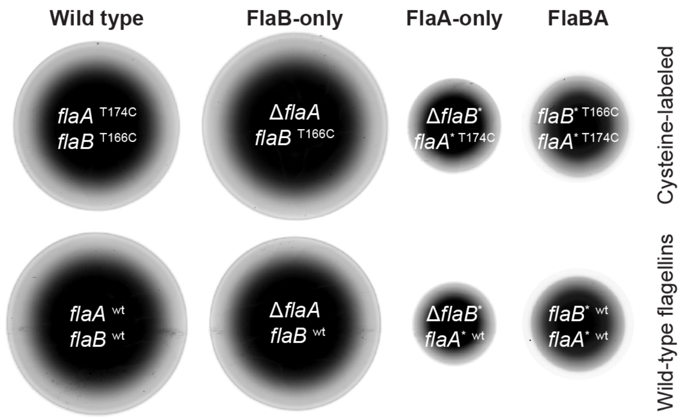

Supplementary Figure 3: Cysteine substitutions in the flagellins do not affect swimming motility. Radial expansion of the different filament type strains with cysteine-labeled and wild-type flagellins was tested in 0.2% soft agar. FlaBA was tested separately so it can only be compared internally. In all strains, including wild type, the lateral flagellins were deleted. The strains depicted in the upper row are also shown in Fig. 1 of the main manuscript, however, as a separate experiment. Asterisks indicate that these genes are expressed from the promoter of the other flagellin.

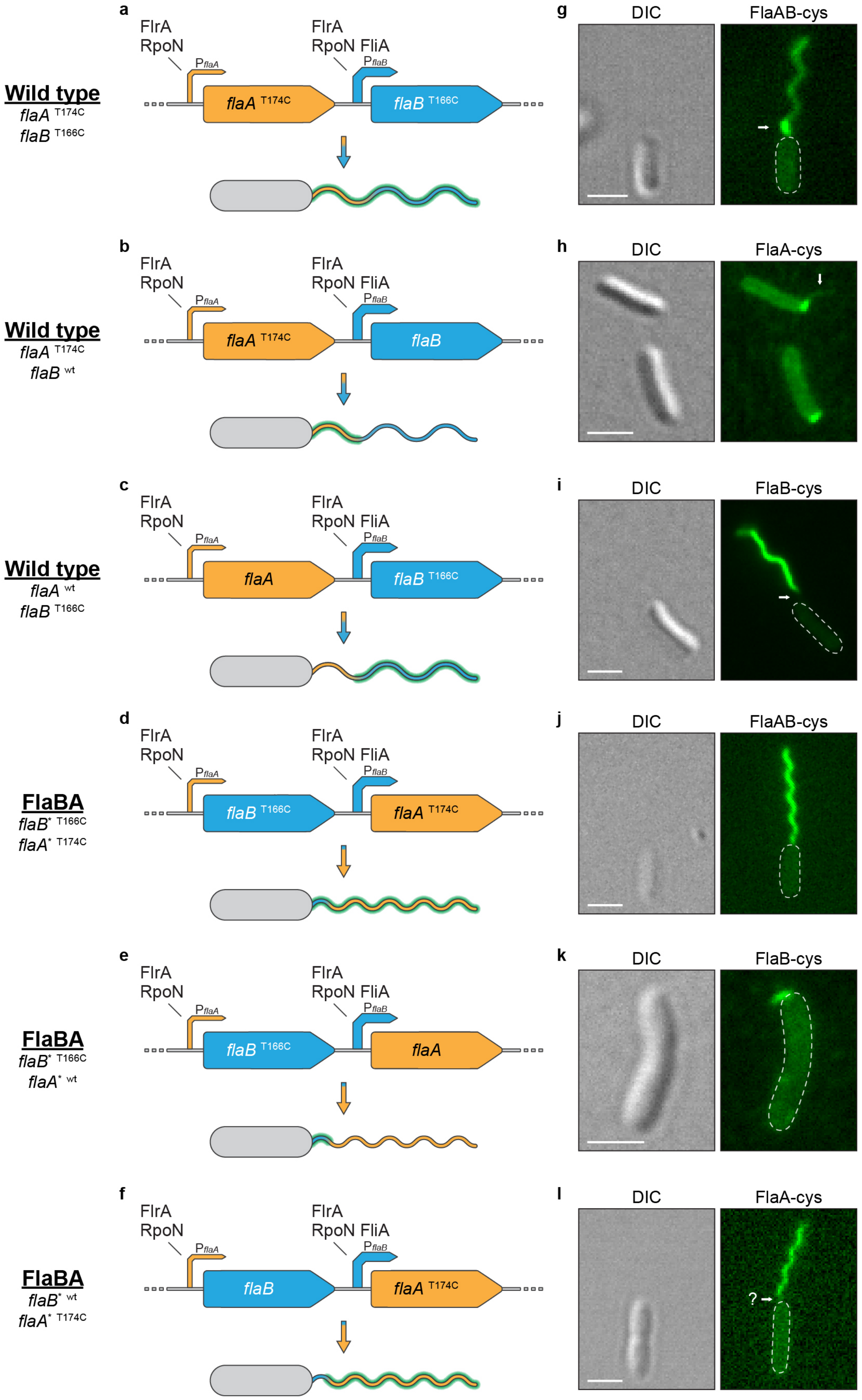

Supplementary Figure 4: Overview of the wild-type and FlaBA filaments essentially as in Fig. 1 of the main text. Additional strains are displayed that produce complete filaments but with only one of the cysteine-labeled flagellins. The glow effect around the filament segment(s) in panels **a** - **f** indicates which of the flagellin segments should be visible in the micrographs of the fluorescently labeled filaments in panels **g** - **l**. The arrows indicate the FlaA portion that is distinguishable from FlaB in the wild type (panel **g**), a faint FlaA signal in the FlaB segment (panel **h**), a gap (unlabeled FlaA) between cell body and the FlaB segment (panel **i**) and the segment where FlaB is expected but not visible in the FlaBA strain (panel **l**), respectively. Scale bars = 2 µm.

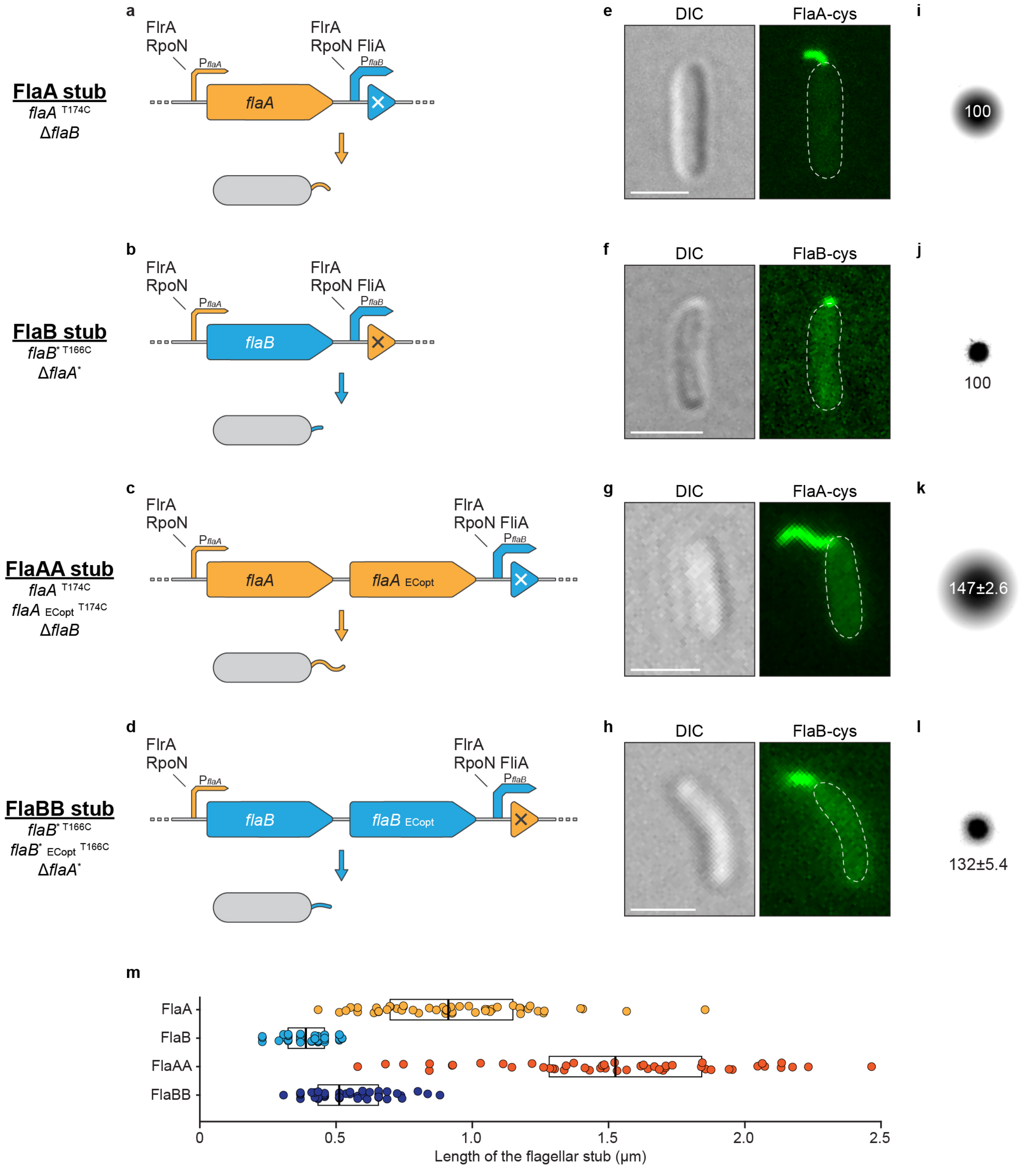

Supplementary Figure 5: Overview of the flagellar filaments that are formed by flagellins under the control of the *flaA* promoter. Lateral flagellin genes were always deleted. **a - d** Genetic organization of the flagellins and modifications to obtain different filaments. The DNA sequences of the duplicated flagellins were codon-optimized for *E. coli* (ECopt) to prevent recombination with the native flagellin genes. Gene deletions are marked with a cross, swapping of the gene sequences is marked with an asterisk. $P_{flaA/B}$ = *flaA*/*B* promoter. **e - h** Micrographs of cells with fluorescently labeled flagellar filaments displaying the outcome of the editing of the flagellin genes. All filaments produced only from the *flaA* promoter form very short stubs. Scale bars = 2 µm. **i - l** Radial expansion in 0.15% soft agar. Strains producing longer filament stubs can spread further. The numbers indicate the relative spreading compared to the corresponding single flagellin strain (% ± s.d.) of three individual experiments (two for FlaBB stub). **m** Length of the filament stubs measured from fluorescently labeled flagellar filaments. FlaB being produced from the *flaA* promoter forms shorter stubs than FlaA being produced from the same promoter. Duplication of a flagellin gene results in increased but not doubled length. Data points are displayed as individual values measured for 50 filaments for each strain. The boxes span the central half of the data points, the black bars indicate the median. Significance was tested for all combinations of all filament stub types. All tested combinations were significant ($P < 0.05$, Bonferroni corrected).

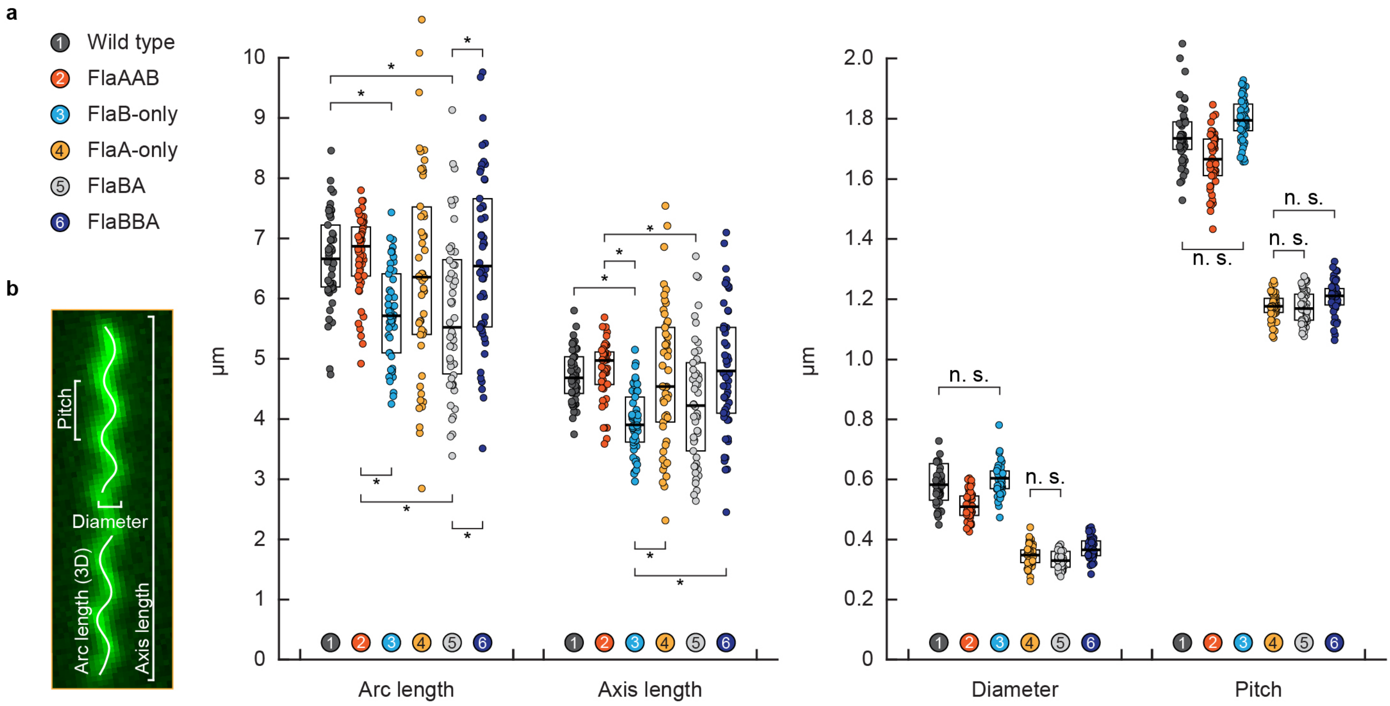

Supplementary Figure 6: Helix geometry of the different flagellar filament types as measured by fluorescent microscopy. **a** The geometric parameters of the flagellar helices are displayed as individual parameters measured for 50 flagellar filaments for each strain. The boxes span the central half of the data points, the black bars indicate the median. The numbered and colored dots indicate which strain is depicted in the diagram. Arc and Axis length show large cell to cell variations but are essentially very similar for all filament types. The three-dimensional arc length was calculated from the axis length, pitch and diameter. Pitch and diameter are very similar for the three filament types mainly or exclusively consisting of FlaB (wild type (FlaAB), FlaAAB and FlaB-only) as well as for the three filament types mainly or exclusively consisting of FlaA (FlaBA, FlaBBA and FlaA-only). Significance was tested for all combinations of filament types for each parameter individually. In the left diagram significant differences ($P < 0.05$, Bonferroni corrected) are indicated by an asterisk. In the right diagram only the non-significant differences are indicated (n.s.). **b** Illustration of which parameters were determined.

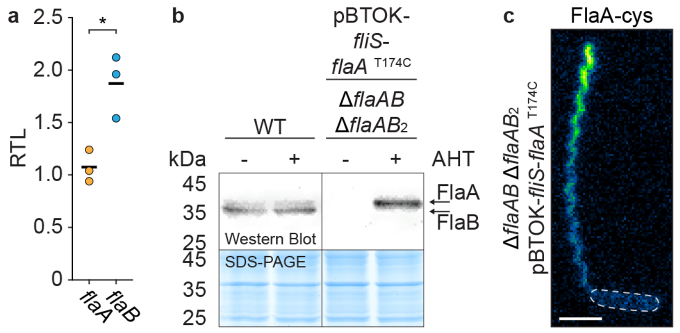

Supplementary Figure 7: Flagellin transcription levels and overexpression of *flaA.* **a** Relative transcript levels (RTL) quantified by qRT-PCR between the flagellin genes *flaA* and *flaB* of the *Shewanella putrefaciens* CN-32 wild-type strain. Transcription of *flaB* is about twice as high as of *flaA*. RTL for the three biological replicates are shown separately (colored dots) together with the mean value (black bar). The differences in RTL are significant ($P < 0.05$), indicated by an asterisk. **b** Immunoblotting analysis (with corresponding Coomassie stained PAGE loading control in the lower panels) of the overexpression of *flaA* (and its chaperone *fliS*) from a plasmid in a background strain lacking polar (*flaAB*) and lateral (*flaAB*$_2$) flagellin genes. The full blot is shown in Supplementary Fig. 13. The wild type without the plasmid (left) produces the native flagellins FlaA and FlaB which can be distinguished by their molecular weight (cp. Supplementary Fig. 2a). In the overexpression strain (right) FlaA is produced from the plasmid with the correct molecular weight, indicating that the post-transcriptional modification is not disturbed (cp. Supplementary Fig. 2a). Expression was induced with anhydrotetracycline (± AHT). This strain forms aberrantly long flagellar filaments only consisting of FlaA (micrograph shown in panel **c**) when induced with the same concentration of AHT as is **b**. The calculated arc length of the displayed flagellum is 18.7 µm. Scale bar = 2 µm.

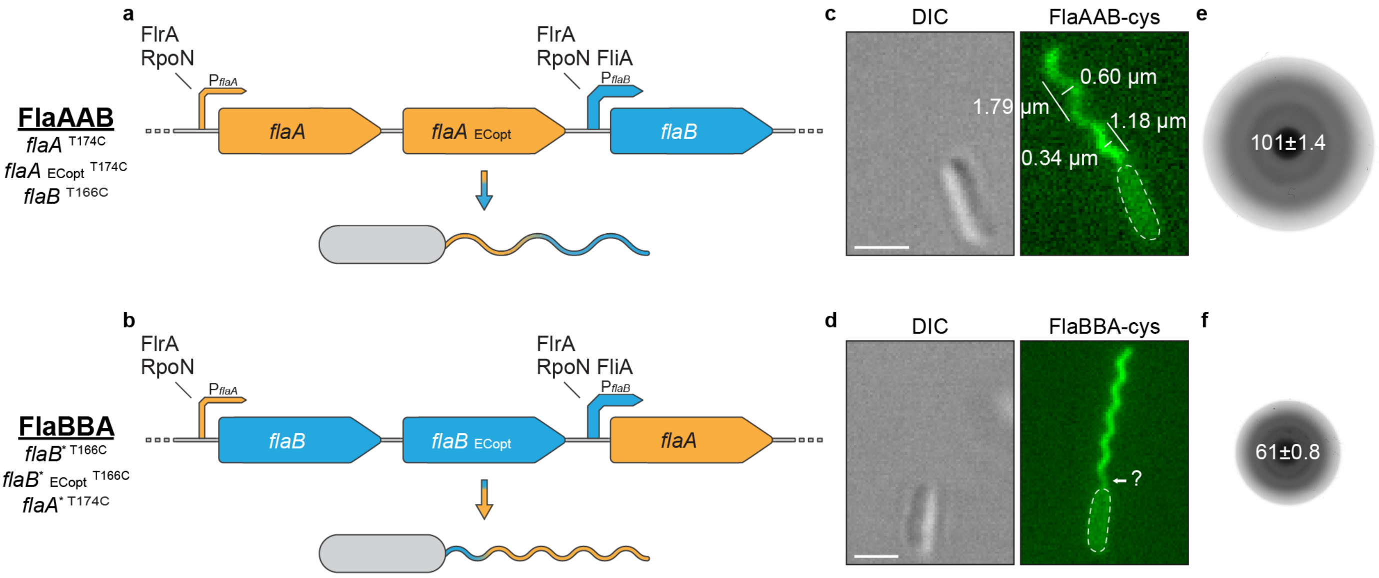

Supplementary Fig. 8: Overview of the flagellin duplication strains. In all strains the genes for the lateral flagellins were deleted. **a, b** Genetic organization of the flagellins and genetic modifications to obtain different filament types. The duplicated genes are both under the control of the *flaA* promoter producing two identical flagellin proteins. The DNA sequences of the synthetic flagellins were codon-optimized for *E. coli* (ECopt) to prevent recombination with the native flagellin genes. Swapping of the gene sequences is marked with an asterisk. $P_{flaA/B}$ = *flaA*/*B* promoter. **c, d** Micrographs of cells with fluorescently labeled flagellar filaments displaying the outcome of the genetic editing of the flagellin genes. Duplication of the upstream flagellin gene increases the length of the proximal flagellin segment, although not doubling it (see also Supplementary Fig. 5). Here, the parameters of the proximal FlaA segment of the FlaAAB filament can be measured as it forms a complete helix turn (numbers in panel **c**). They fit well to the parameters of the FlaA-only filament, while the parameters of the remaining major segment fit well to the parameters of the FlaB-only filament (cp. Supplementary Fig. 6 and Supplementary Table 4). The proximal FlaB segment of the FlaBBA filament is still not clearly distinguishable from the cell body and the major part of the flagellum (panel **d**, question mark). Scale bars represent 2 µm. **e, f** Radial expansion of the flagellin duplication cells in 0.25% soft agar. The numbers indicate the relative spreading compared to the wild type (% ± s.d.) of three individual experiments (cp. Fig. 1i in the main manuscript). The FlaAAB strain performs comparatively well as the wild type and FlaB-only strains, while the FlaBBA strain spreads comparatively to the FlaBA and FlaA-only strains.

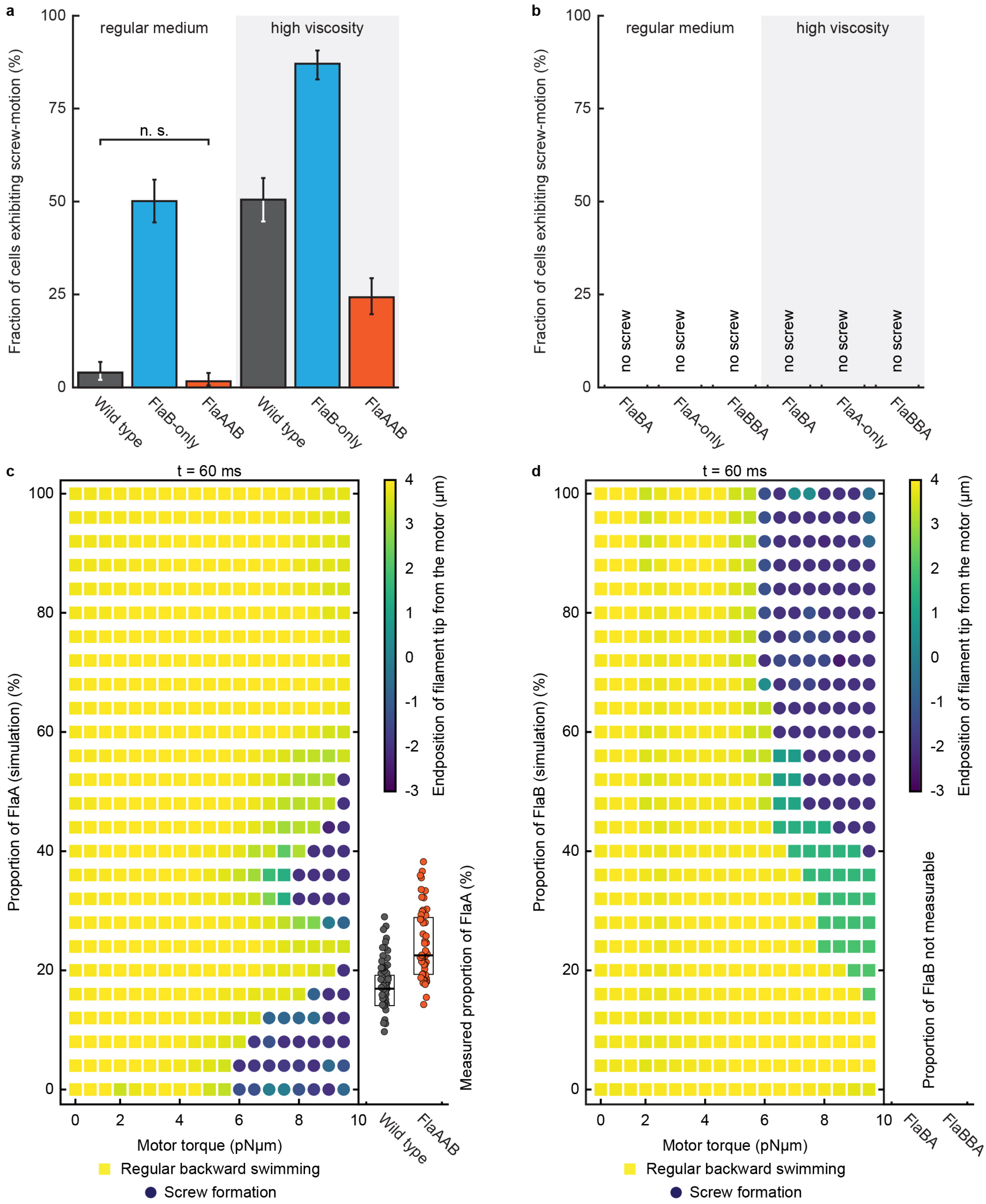

Supplementary Figure 9: Experimentally observed screw formation and simulation of screw formation. **a** and **c** are the same data as in Fig. 4 of the main text together with the flagellin duplication strains for comparison. **a, b** Screw formation observed for different filaments in regular and high viscosity. Significance was tested for all filament type combinations under both conditions and for each filament type between the two conditions. If no screw was observed at all, significance was not tested. Only the difference between wild type (FlaAB) and FlaAAB filament cells in regular medium is not significant (n.s.; $P < 0.05$, Bonferroni corrected). Error bars indicate 95% confidence intervals. About 300 cells were counted for each strain. **c, d** Observation of screw formation for varying flagellin compositions after a simulation time of t = 60 ms. The diagram in panel **c** is identical with the diagram in Fig. 4b. The simulation data displayed in panel **d** was obtained similarly but this time starting with a flagellum completely composed of FlaA (bottom of the diagram) and successively exchanging the segments to a FlaB configuration starting from the filament's base at the cell pole. The formation of a screw is indicated by blue circles. The color coding represents the z-position of the flagellum's free end, with negative values indicating a position below the motor segment (position 0). Blue-green squares therefore indicate filament instabilities that are, however, not comparable to proper screw formation.

Supplementary Figure 10: The flagellins of *Shewanella oneidensis* MR-1 show a similar spatial distribution as the polar flagellins of *Shewanella putrefaciens* CN-32 in the assembled flagellum. **a** Micrograph of FlaA-cysteine-labeled cells. **b** Micrograph of FlaB-cysteine-labeled cells. The arrow indicates a gap (unlabeled FlaA) between cell body and the FlaB segment. Scale bars represent 2 µm.

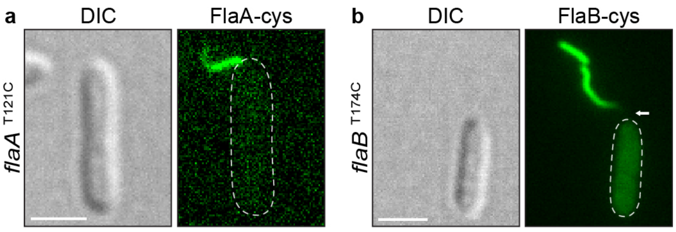

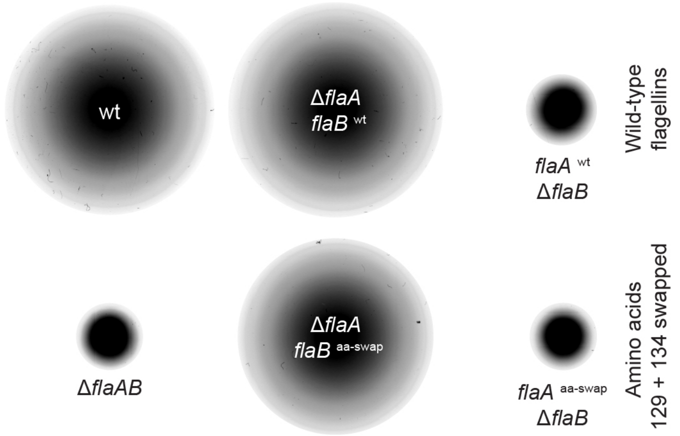

Supplementary Figure 11: Exchanging the amino acids of the flagellins FlaA and FlaB at the positions 129 and 134 does not affect the swimming ability of *Shewanella putrefaciens* CN-32 (here: radial expansion in 0.3% soft agar). It was proposed elsewhere that the corresponding homologous amino acids in *Shewanella oneidensis* MR-1 are responsible for the functional differences of the two flagellins [1]. The effect was analyzed in strains only expressing one of the two polar flagellins and both lateral flagellins.

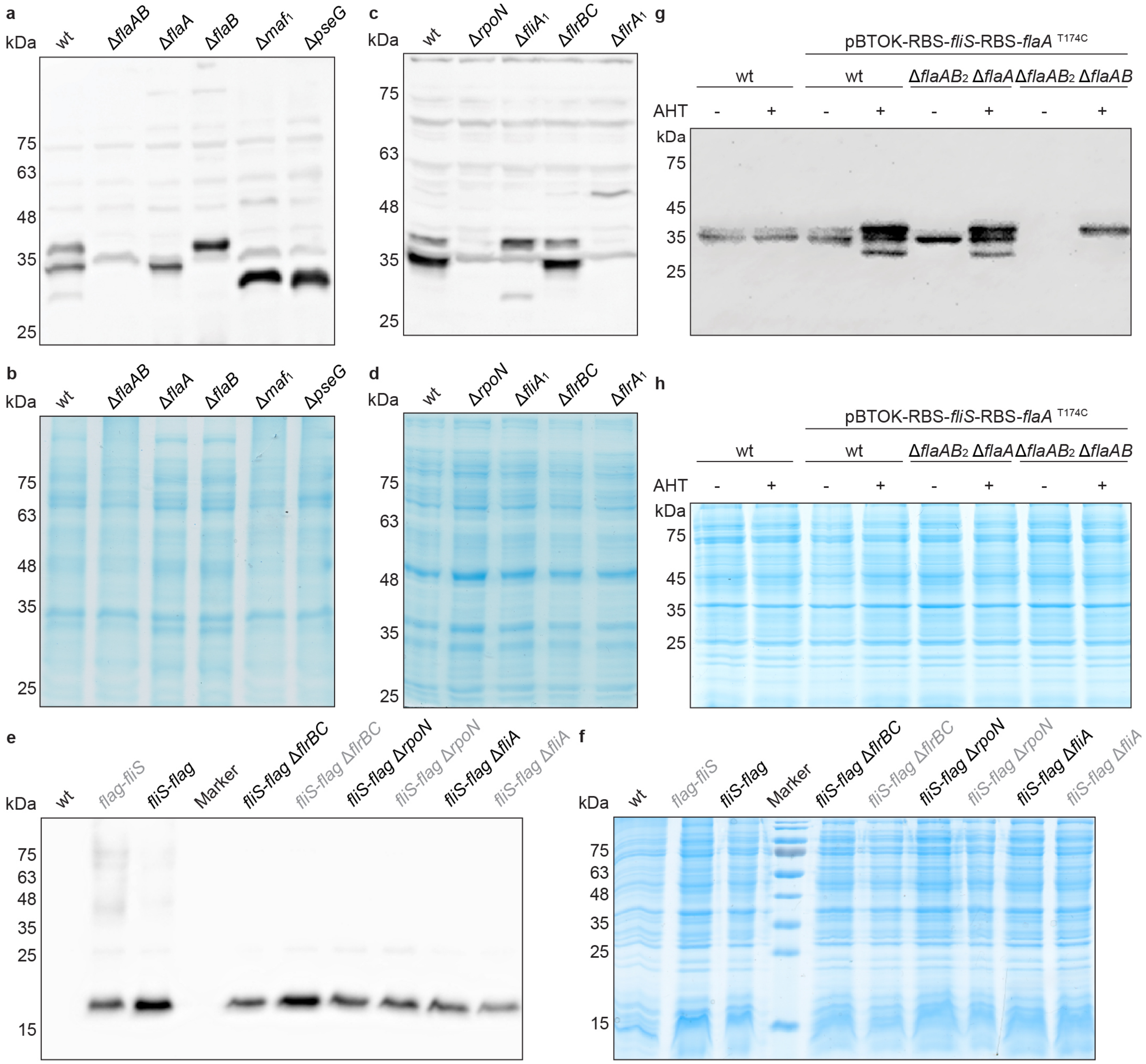

Supplementary Figure 12: Full immunoblots and Coomassie-stained SDS-PAGE gels. **a** Blot and **b** gel of Supplementary Fig. 2a. **c** Blot and **d** gel of Supplementary Fig. 2b. **e** Blot and **f** gel of Supplementary Fig. 2c. Strains marked in grey were backup strains that were not used furthermore. Marker = MARKER VI Pre-colored, AppliChem. **g** Blot and **h** gel of Supplementary Fig. 7b. Expression was induced with anhydrotetracycline (± AHT).

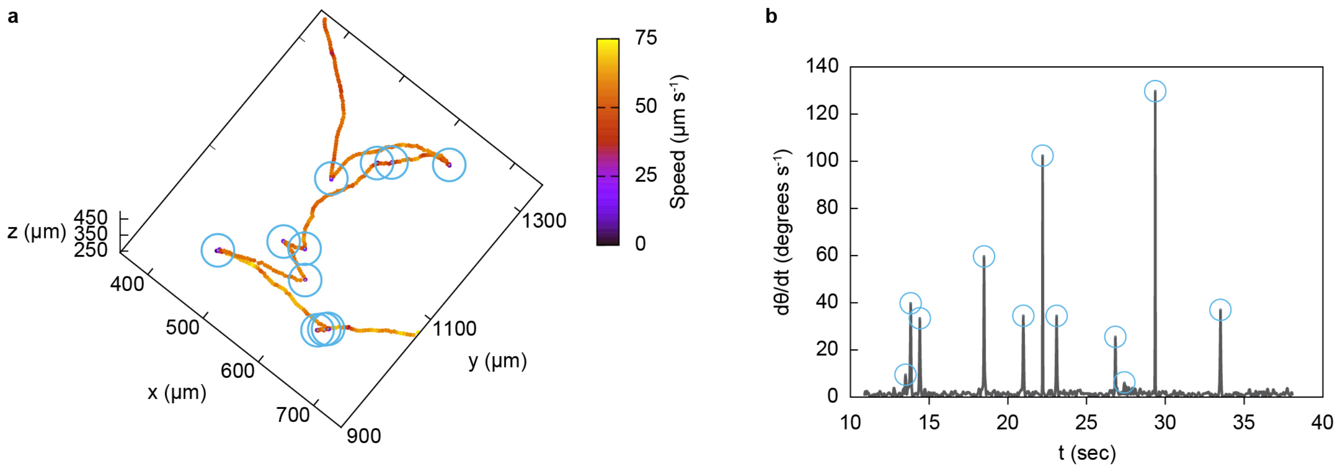

Supplementary Figure 13: Example data of a cell track from the holographic cell tracking. **a** A nearly top-down view of the cell track shown in Fig. 3j of the main manuscript. Swimming speed is encoded by color according to the color bar on the right, and the re-orientation events are highlighted with blue circles. **b** A graph of the cell's change in swimming direction, dθ/dt, as a function of time. Reorientation events are marked by blue circles, at points where dθ/dt rises above a threshold level of 5 degrees per second.

Supplementary Tables

Supplementary Table 1: Bacterial strains

Identifier	Relevant genotype	Purpose	Reference
Escherichia coli			
DH5α λ*pir*	ϕ80d*lacZ* ΔM15 Δ(*lacZYA-argF*)U169 *recA*$_1$ *hsdR17 deoR thi-l supE44 gyrA96 relA*$_1$/λ*pir*	cloning strain	2
WM3064	*thrB1004 pro thi rpsL hsdS lacZ* ΔM15 RP4-1360 Δ(*araBAD*) 567Δ*dapA* 1341::[*erm pir*(wt)]	conjugation strain	3
Shewanella putrefaciens			
S271	CN-32	wild type	4
S2576	Δ*flaAB*$_2$ (Sputcn32_3455-3456)	markerless deletion of both lateral flagellin genes	5
S2575	Δ*flaAB*$_1$ (Sputcn32_2585-2586)	markerless deletion of both polar flagellin genes	5
S4433	Δ*flaAB*$_1$; Δ*flaAB*$_2$	markerless deletion of both polar and both lateral flagellin genes	this study
S3807	Δ*flaA*$_1$-ext (Sputcn32_2586)	markerless deletion of polar minor flagellin gene; extended knock-out	6
S3810	Δ*flaB*$_1$-ext (Sputcn32_2585)	markerless deletion of polar major flagellin gene; extended knock-out	6
S5179	Δ*flaB*$_1$; Δ*flaAB*$_2$	markerless deletion of polar major flagellin gene and both lateral flagellin genes	this study
S4387	Δ*flaA*$_1$; Δ*flaAB*$_2$	markerless deletion of polar minor flagellin gene and both lateral flagellin genes	this study
S5165	Δ*flaB*$_1$; *flaA*$_1$ (positions swapped); Δ*flaAB*$_2$	markerless insertion of position swapped wild-type *flaA*$_1$ gene and Δ*flaB*$_1$ into Δ*flaAB*$_1$ and deletion of lateral flagellin genes	this study
S4151	*flaA*$_1$-S169C	markerless insertion of cysteine-labeled *flaA*$_1$ gene into Δ*flaA*$_1$-ext	this study
S4152	*flaA*$_1$-T174C; Δ*flaAB*$_2$	markerless insertion of cysteine-labeled *flaA*$_1$ gene into Δ*flaA*$_1$-ext and deletion of lateral flagellin genes	6
S4143	*flaB*$_1$-T166C	markerless insertion of cysteine-labeled *flaB*$_1$ gene into Δ*flaB*$_1$-ext	6
S4154	*flaB*$_1$-T166C; Δ*flaAB*$_2$	markerless insertion of cysteine-labeled *flaB*$_1$ gene into Δ*flaB*$_1$-ext and deletion of lateral flagellin genes	6
S4352 **FlaB-only**	Δ*flaA*$_1$; *flaB*$_1$-T166C; Δ*flaAB*$_2$	markerless insertion of cysteine-labeled *flaB*$_1$ gene into Δ*flaB*$_1$-ext and markerless deletion of *flaA*$_1$ and Δ*flaAB*$_2$	this study

S4401 **Wild type**	*flaA*$_1$-T174C; *flaB*$_1$-T166C; Δ*flaAB*$_2$	markerless insertion of cysteine-labeled *flaB*$_1$ gene into Δ*flaB*$_1$-ext and cysteine-labeled *flaA*$_1$ gene into Δ*flaA*$_1$-ext and deletion of lateral flagellin genes	6
S4794 **FlaA-only**	Δ*flaB*$_1$; *flaA*$_1$-T174C (positions swapped); Δ*flaAB*$_2$	markerless insertion of position swapped, cysteine-labeled *flaA*$_1$ gene and Δ*flaB*$_1$ into Δ*flaAB*$_1$ and deletion of lateral flagellin genes	this study
S4795 **FlaB stub**	*flaB*$_1$*-T166C;* Δ*flaA*$_1$ (positions swapped); Δ*flaAB*$_2$	markerless insertion of position swapped, cysteine-labeled *flaA*$_1$ gene and Δ*flaB*$_1$ into Δ*flaAB*$_1$ and deletion of lateral flagellin genes	this study
S5219 **FlaA stub**	*flaA*$_1$-T174C; Δ*flaB*$_1$; Δ*flaAB*$_2$	markerless insertion of cysteine-labeled *flaA*$_1$ gene into Δ*flaA*$_1$-ext and deletion of deletion of polar major flagellin gene and lateral flagellin genes	this study
S5538	*flaB*$_1$; *flaA*$_1$-T174C (positions swapped); Δ*flaAB*$_2$	markerless insertion of wild-type *flaB*$_1$ gene downstream of the *flaA*$_1$ promoter into S4794	this study
S5539 **FlaBA**	*flaB*$_1$*-T166C*; *flaA*$_1$-T174C (positions swapped); Δ*flaAB*$_2$	markerless insertion of cysteine-labeled *flaB*$_1$ gene downstream of the *flaA*$_1$ promoter into S4794	this study
S5705 **FlaAAB**	*flaA*$_1$-T174C; *flaA*$_1$-T174C (*E. coli* optimized); *flaB*$_1$-T166C; Δ*flagAB*$_2$	markerless insertion of cysteine-labeled *flaA*$_1$ and an additional *E. coli* optimized *flaA*$_1$ into S4352	this study
S5706 **FlaAA stub**	*flaA*$_1$-T174C; *flaA*$_1$-T174C (*E. coli* optimized); Δ*flaB*$_1$; Δ*flagAB*$_2$	markerless insertion of cysteine-labeled *flaA*$_1$ and an additional *E. coli* optimized *flaA*$_1$ and *ΔflaB*$_1$ into S4433	this study
S5707 **FlaBBA**	*flaB*$_1$-T166C; *flaB*$_1$-T166C (*E. coli* optimized); *flaA*$_1$-T174C (positions swapped); Δ*flagAB*$_2$	markerless insertion of cysteine-labeled *flaB*$_1$ and an additional *E. coli* optimized *flaB*$_1$ downstream of the *flaA*$_1$ promoter into S4794	this study
S5708 **FlaBB stub**	*flaB*$_1$-T166C; *flaB*$_1$-T166C (*E. coli* optimized); Δ*flaA*$_1$ (positions swapped); Δ*flagAB*$_2$	markerless insertion of cysteine-labeled *flaB*$_1$ and an additional *E. coli* optimized *flaB*$_1$ downstream of the *flaA*$_1$ promoter and *ΔflaA*$_1$ into S4433	this study
S3142	Δ*rpoN* (Sputcn32_0715)	markerless deletion of the *pseG* gene (sigma factor 54)	this study
S2673	Δ*fliA*$_1$ (Sputcn32_2559)	markerless deletion of the *fliA*$_1$ gene (sigma factor 28)	this study
S3139	Δ*flrA*$_1$ (Sputcn32_2580)	markerless deletion of the *flrA*$_1$ gene	this study
S3174	Δ*flrBC* (Sputcn32_2578-2579)	markerless deletion of the *flrB* and *flrC* genes	this study
S3864	Δ*maf1* (Sputcn32_2630)	markerless deletion of the *maf1* gene	this study
S3863	Δ*pseG* (Sputcn32_2626)	markerless deletion of the *pseG* gene	this study
S4440	pBTOK-RBS-*fliS*$_1$-RBS-*flaA*$_1$-T174C	stable integration of overproduction vector pBTOK producing the full length proteins FliS$_1$ and FlaA$_1$	this study

S4441	Δ*flaA*$_1$; Δ*flaAB*$_2$ -- pBTOK-RBS-*fliS*$_1$-RBS-*flaA*$_1$-T174C	markerless deletion of polar minor flagellin gene and both lateral flagellin genes and stable integration of overproduction vector pBTOK producing the full length proteins FliS$_1$ and FlaA$_1$	this study
S4442	Δ*flaAB*$_1$; Δ*flaAB*$_2$ -- pBTOK-RBS-*fliS*$_1$-RBS-*flaA*$_1$-T174C	markerless deletion of both polar flagellin genes and both lateral flagellin genes and stable integration of overproduction vector pBTOK producing the full length proteins FliS$_1$ and FlaA$_1$	this study
S3127	Δ*fliS*$_1$ (Sputcn32_2581)	markerless deletion of the *fliS*$_1$ gene	this study
S4394	*fliS*$_1$-FLAG	markerless insertion of the *fliS*$_1$ gene with a c-terminal FLAG-tag	this study
S3791	Δ*flaB*$_1$; *flaA*$_1$-S129N-N134T	markerless insertion of amino-acid-swapped *flaA*$_1$ gene into Δ*flaA*$_1$ and deletion of polar major flagellin gene	this study
S3792	Δ*flaA*$_1$; *flaB*$_1$-N129T-T134N	markerless insertion of amino-acid-swapped *flaB*$_1$ gene into Δ*flaB*$_1$ and deletion of polar major flagellin gene	this study

Shewanella oneidensis

S565	MR-1	wild type	7
S1021	Δ*flaA* (SO_3238)	markerless deletion of polar minor flagellin gene	8
S1020	Δ*flaB* (SO_3237)	markerless deletion of polar major flagellin gene	8
S4858	*flaA*-T121C	markerless insertion of cysteine-labeled *flaA* gene into Δ*flaA*	this study
S4857	*flaB*-S174C	markerless insertion of cysteine-labeled *flaB* gene into Δ*flaB*	this study

Supplementary Table 2: Plasmids

**Name**	**Insert**	**Purpose**	**Reference**
pNPTS138-R6KT	*mob*RP4+ *ori*-R6K *sacB* β-galactosidase fragment alpha Km^r	suicide plasmid for in-frame deletions or integrations	9
pNPTS138-R6KT-flag-clusterI-KO	Δ*flaAB*$_1$ (Sputcn32_ 2585-2586)	in-frame deletion fragment	5
pNPTS138-R6KT-flag-clusterII-KO	Δ*flaAB*$_2$ (Sputcn32_ 3455-3456)	in-frame deletion fragment	5
pNPTS138-R6KT-*flaA*$_1$-KO-ext	Δ*flaA*$_1$ (Sputcn32_2586)	in-frame deletion fragment	6
pNPTS138-R6KT-*flaB*$_1$-KO-ext	Δ*flaB*$_1$ (Sputcn32_2585)	in-frame deletion fragment	6
pNPTS138-R6KT-*flaA*$_1$-S169C	*flaA*$_1$-S169C (Sputcn32_2586)	in-frame insertion fragment; serine 169 substituted with cysteine	this study
pNPTS138-R6KT-*flaA*$_1$-T174C	*flaA*$_1$-T174C (Sputcn32_2586)	in-frame insertion fragment; threonine 174 substituted with cysteine	6
pNPTS138-R6KT-*flaB*$_1$-T166C	*flaB*$_1$-T166C (Sputcn32_2585)	in-frame insertion fragment, threonine 166 substituted with cysteine	6

pNPTS138-R6KT-*flaB*$_1$-S174C	*flaB*$_1$-S174C (Sputcn32_2585)	in-frame insertion fragment; serine 174 substituted with cysteine	6
pNPTS138-R6KT-*flaB*$_1$-KO - *flaA*$_1$-T174C (positions swapped)	Δ*flaB*$_1$ (Sputcn32_2585) - flaA$_1$-T174C (Sputcn32_2586)	in-frame insertion fragment; Δ*flaB*$_1$ replaces *flaA*$_1$ and *flaA*$_1$ replaces *flaB*$_1$; threonine 174 of *flaA*$_1$ substituted with cysteine	this study
pNPTS138-R6KT-*flaB*$_1$-T166C - *flaA*$_1$-KO (positions swapped)	*flaB*$_1$-T166C (Sputcn32_2585) - ΔflaA$_1$ (Sputcn32_2586)	in-frame insertion fragment; *flaB*$_1$ replaces *flaA*$_1$ and Δ*flaA*$_1$ replaces *flaB*$_1$; threonine 166 of *flaB*$_1$ substituted with cysteine	this study
pNPTS138-R6KT-*flaB*$_1$-KO - *flaA*$_1$ (positions swapped)	Δ*flaB*$_1$ (Sputcn32_2585) - flaA$_1$ (Sputcn32_2586)	in-frame insertion fragment; Δ*flaB*$_1$ replaces *flaA*$_1$ and *flaA*$_1$ replaces *flaB*$_1$	this study
pNPTS138-R6KT-*flaB*$_1$ (positions swapped)	*flaB*$_1$-T166C (Sputcn32_2585)	in-frame insertion fragment; *flaB*$_1$ replaces deletion fragment of *flaA*$_1$	this study
pNPTS138-R6KT-*flaB*$_1$-T166C (positions swapped)	*flaB*$_1$-T166C (Sputcn32_2585)	in-frame insertion fragment; *flaB*$_1$ replaces deletion fragment of *flaA*$_1$; threonine 166 of *flaB*$_1$ substituted with cysteine	this study
puc57-KAN-*flaA*$_1$ (*E. coli* optimized)	*flaA*$_1$ (*E. coli* optimized)	synthetic *flaA*$_1$ of *Shewanella putrefaciens* CN-32 codon optimized for *Escherichia coli* K-12 to prevent recombination with the native *flaA*$_1$	this study
puc57-KAN-*flaB*$_1$ (*E. coli* optimized)	*flaB*$_1$ (*E. coli* optimized)	synthetic *flaB*$_1$ of *Shewanella putrefaciens* CN-32 codon optimized for *Escherichia coli* K-12 to prevent recombination with the native *flaB*$_1$	this study
pNPTS138-R6KT-double-*flaA*$_1$ - for *ΔflaA*$_1$	*flaA*$_1$-T174C; *flaA*$_1$-T174C (*E. coli* optimized)	in-frame insertion fragment; *flaA*$_1$ and an additional *E. coli* optimized *flaA*$_1$ replace the deletion fragment of *flaA*$_1$; threonine 174 of both *flaA*$_1$ genes substituted with cysteine	this study
pNPTS138-R6KT-double-*flaA*$_1$ - for *ΔflaAB*$_1$	*flaA*$_1$-T174C; *flaA*$_1$-T174C (*E. coli* optimized); Δ*flaB*$_1$	in-frame insertion fragment; *flaA*$_1$ and an additional *E. coli* optimized *flaA*$_1$ and deletion of *flaB*$_1$ replace the deletion fragment of *flaAB*$_1$; threonine 174 of both *flaA*$_1$ genes substituted with cysteine	this study
pNPTS138-R6KT-double-*flaB*$_1$ - for *ΔflaB*$_1$ *flaA*$_1$ (position swapped)	*flaB*$_1$-T166C; *flaB*$_1$-T166C (*E. coli* optimized) (positions swapped)	in-frame insertion fragment; *flaB*$_1$ and an additional *E. coli* optimized *flaB*$_1$ replace the position swapped deletion fragment of *flaB*$_1$; threonine 166 of both *flaB*$_1$ genes substituted with cysteine	this study
pNPTS138-R6KT-double-*flaA*$_1$ - for *ΔflaA*$_1$	*flaB*$_1$-T166C; *flaB*$_1$-T166C (*E. coli* optimized); Δ*flaA*$_1$ (positions swapped)	in-frame insertion fragment; *flaB*$_1$ and an additional *E. coli* optimized *flaB*$_1$ and deletion of *flaA*$_1$ (position swapped) replace the deletion fragment of *flaAB*$_1$; threonine 166 of both *flaB*$_1$ genes substituted with cysteine	this study

pNPTS138-R6KT-*rpoN*-KO	Δ*rpoN* (Sputcn32_0715)	in-frame deletion fragment	this study
pNPTS138-R6KT-*fliA*$_1$-KO	Δ*fliA*$_1$ (Sputcn32_2559)	in-frame deletion fragment	this study
pNPTS138-R6KT-*flrA*$_1$-KO	Δ*flrA*$_1$ (Sputcn32_2580)	in-frame deletion fragment	this study
pNPTS138-R6KT-*flrBC*-KO	Δ*flrBC* (Sputcn32_2578-79)	in-frame deletion fragment	this study
pNPTS138-R6KT-*maf1*-KO	Δ*maf1* (Sputcn32_2630)	in-frame deletion fragment	this study
pNPTS138-R6KT-*pseG*-KO	Δ*pseG* (Sputcn32_2626)	in-frame deletion fragment	this study
pNPTS138-R6KT-*fliS*$_1$-KO	Δ*fliS*$_1$ (Sputcn32_2581)	in-frame deletion fragment	this study
pNPTS138-R6KT-*fliS*$_1$-FLAG	*fliS*$_1$-FLAG (Sputcn32_2581)	in-frame insertion fragment; c-terminal FLAG-tagged version of *fliS*$_1$	this study
pNPTS138-R6KT-*flaA*$_1$-S129N-N134T	*flaA*$_1$-S129N-N134T (Sputcn32_2586)	in-frame insertion fragment; amino acid swap with *flaB*$_1$	this study
pNPTS138-R6KT-*flaB*$_1$-N129T-T134N	*flaB*$_1$-N129T-T134N (Sputcn32_2581)	in-frame insertion fragment; amino acid swap with *flaA*$_1$	this study
pBTOK	pBBR1-MCS2 backbone (pBBR origin, Km^r); TetR, Promoter and multiple cloning site of pASK-IBA3plus and *E. coli* rrnB1 T1 and lambda phage T0 terminator	overproduction plasmid inducible with anhydrotetracycline	10
pBTOK-RBS-*fliS*$_1$-RBS-*flaA*$_1$-T174C	RBS-*fliS*$_1$-(Sputcn32_2581) - RBS-*flaA*$_1$-T174C (Sputcn32_2586)	overproduction of wild-type *fliS*$_1$ and cysteine-labeled *flaA*$_1$, both with the ribosome binding site sequence AGGAGG; threonine 174 of *flaA*$_1$ substituted with cysteine	this study

Supplementary Table 3: Oligonucleotides

#	Name	Sequence 5'-3'	Purpose
B 31	BamHI-flagL-fwd	AGG ATC CTG ACA CTG TAT TTA TGG CGC AGG	construction of in-frame deletion vector pNPTS138-R6KT-R6KT-flag-clusterII-KO
B 32	OL-flagL-rev	CAG TAG ACC GTG AAC ACC TAA CAT ATT AAT TCT CCA G	
B 33	OL-flagL-fwd	GGT GTT CAC GGT CTA CTG CGT TAA TCT AGC TC	
B 34	PspOMI-flagL-rev	TGT CGG GCC CGT CGC CGT CGC ATT TTC GC	
B 35	Check-flagL-fwd	GTA TTA GCT TCG ATC GGG ATT GG	check primer for flaAB$_2$
B 36	Check-flagL-rev	GTT ACC CTT TGG CGC ATC GG	
B 45	EcoRI-flagP-fwd	A GAA TTC GAA GTT AAA GTG TCT GGG AAA CCC	construction of in-frame deletion vector
B 46	OL-flagP-rev	TCA CCT CTT AAC TGT AAT AGC CAT AGT ATT TTC CTC	

B 47	OL-flagP-fwd	ATT ACA GTT AAG AGG TGA GAC AGT GAT AGG GA	pNPTS138-R6KT-R6KT-flag-clusterI-KO
B 48	PspOMI-flagP-rev	T CTA GGG CCC TAA GCC TCT GTT TTC ATC AAA AGC C	
B 49	Check-flagP-fwd	AAT TTT GAT GCG ACT ACC CCC G	check primer for *flaAB*$_1$
B 50	Check-flagP-rev	TAT CTA GAC CTG ACC CCA TGC C	
MJK 26	OL_flaA1_KO_ext_rv	GTG ACA GCG CAA TAG CCA TAG TAT TTT CCT CTT CTA AG	construction of in-frame deletion vector pNPTS138-R6KT-*flaA*$_1$-KO-ext
MJK 27	OL_flaA1_KO_ext_fw	TAT GGC TAT TGC GCT GTC ACT ACT GGG ATA ATT TAC	
FR 299	EcoRV_flaA1_KO_fw	GAA TTC GTG GAT CCA GAT GAA GTT AAA GTG TCT GGG AAA CCC	
FR 302	EcoRV_flaA1_KO_rv	CAA GCT TCT CTG CAG GAT GCA TCG CAC CTT CAG AAA TTT GG	
MJK 28	OL_flaB1_KO_ext_rv	AGG CCA CTT GGG CCA TGA TCG TTT CCT CTG TA	construction of in-frame deletion vector pNPTS138-R6KT-*flaB*$_1$-KO-ext
MJK 29	OL_flaB1_KO_ext_fw	GAT CAT GGC CCA AGT GGC CTT ATC ACT GCT GTA ATA G	
FR 295	EcoRV_flaB1_KO_fw	GAA TTC GTG GAT CCA GAT ATA ACC AAC GTG CAG CGT TAG G	
FR 298	EcoRV_flaB1_KO_rv	CAA GCT TCT CTG CAG GAT CAG CTA ATG CCA ACG CTT CTT C	
MJK 76	FlaA1-S169C-R	CCA TTA AAC ACC CCG CAT TAT TGG TAC C	construction of in-frame insertion vector pNPTS138-R6KT-*flaA*$_1$-S169C
MJK 77	FlaA1-S169C-F	ATG CGG GGT GTT TAA TGG TTA GTA CAT TAA CG	
MJK 78	FlaA1-T174C-R	TCG TTA AAC AAC TAA CCA TTA AAC TCC CCG CAT TAT TGG	construction of in-frame insertion vector pNPTS138-R6KT-*flaA*$_1$-T174C
MJK 79	FlaA1-T174C-F	TGG TTA GTT GTT TAA CGA TTG CAA CTT CAG GTG GTC G	
MJK 82	FlaB1-T166C-R	CTG ATG CAC AGG TTT TTG ACA CAG AAA TCG	construction of in-frame insertion vector pNPTS138-R6KT-*flaB*$_1$-T166C
MJK 83	FlaB1-T166C-F	CAA AAA CCT GTG CAT CAG CAT TAA AAG TTG G	
MJK 86	FlaB1-S174C-R	ATA TCT AAA CAA CCA ACT TTT AAT GCT GAT GC	construction of in-frame insertion vector pNPTS138-R6KT-flaB1-S174C
MJK 87	FlaB1-S174C-F	AGT TGG TTG TTT AGA TAT TAA AGG CTC TGC	
MJK 186	A1us-oeB1ko-R	GCA GTG ATA AGG CCA CTT GGG CCA TAG TAT TTT CCT CTT CTA AGT ATC TTG C	construction of in-frame insertion vectors pNPTS138-R6KT-*flaB*-KO - *flaA*$_1$ (positions swapped) and pNPTS138-R6KT-*flaB*$_1$-KO - *flaA*$_1$-T174C (positions swapped)
MJK 187	A1ds-oeB1ko-F	CCA AGT GGC CTT ATC ACT GCT GTA ATT TAC TAA GCA GAA CAT ATC AAT ACA GGA CGT TGC	
MJK 188	B1us-oeA1-R	TAA TAG CCA TGA TCG TTT CCT CTG TAT ACC	
MJK 189	A1-oeB1us-F	GGA AAC GAT CAT GGC TAT TAC AGT TAA TAC CAA CGT GAC	
MJK 190	A1-oeB1ds-R	TTG CTT TCT ATT ATC CCA GTA GTG ACA GCG C	
MJK 191	B1ds-oeA1-F	ACT GGG ATA ATA GAA AGC AAG AGG TGA GAC AGT G	

MJK 180	A1us-oeB1_R	TAA TGG CCA TAG TAT TTT CCT CTT CTA AGT ATC TTG C	construction of in-frame insertion vector pNPTS138-R6KT-*flaB*$_1$-T166C - *flaA*$_1$-KO (positions swapped)
MJK 181	B1-oeA1us_F	GGA AAA TAC TAT GGC CAT TAC AGT AAA CAC TAA CG	
MJK 182	B1-oeA1ds_R	GCT TAG TAA ATT ACA GCA GTG ATA AGG CCA C	
MJK 183	dsA1-oeB1_F	ACT GCT GTA ATT TAC TAA GCA GAA CAT ATC AAT ACA GGA CGT TGC	
MJK 184	usB1-oeA1ko_R	CCA GTA GTG ACA GCG CAA TAG CCA TGA TCG TTT CCT CTG TAT ACC	
MJK 185	B1ds-oeA1ko_F	TAT TGC GCT GTC ACT ACT GGG ATA ATA GAA AGC AAG AGG TGA GAC AGT GAT AGG	
MJK 258	OL_FlaB_PS_R	GTG TTT ACT GTA ATG GCC ATA GTA TTT TCC TCT TCT AAG TAT C	construction of in-frame insertion vectors pNPTS138-R6KT-*flaB*$_1$ (positions swapped) and pNPTS138-R6KT-*flaB*$_1$-T166C (positions swapped)
MJK 259	FlaB_F	ATG GCC ATT ACA GTA AAC ACT AAC G	
MJK 260	FlaB_R	TTA CAG CAG TGA TAA GGC CAC TTG	
MJK 261	OL_FlaB_PS_F	CAA GTG GCC TTA TCA CTG CTG TAA TTT AC	
MJK 262	Eco_FlaB_PS_R	GCC AAG CTT CTC TGC AGG ATT TGC ACC TTC AGC GAT CTG GG	
MJK 270	OL_FlaA-A_R	ATT TGC TTC CTC TTC TAA GAA TCG AAT TAT CCC AGT AGT GAC AGC GC	construction of in-frame insertion vectors pNPTS138-R6KT-double-*flaA*$_1$ - for Δ*flaA*$_1$ and pNPTS138-R6KT-double-*flaA*$_1$ - for Δ*flaAB*$_1$
MJK 271	OL_A-FlaA_F	TTC GAT TCT TAG AAG AGG AAG CAA ATA TGG CTA TTA CGG TTA ACA CGA ATG TAA CAT C	
MJK 272	OL_A-HOM_R	GAT ATG TTC TGC TTA GTA AAT TAG CCC AGA AGC GAC AGC G	
MJK 273	OL_HOM-A_F	TTT ACT AAG CAG AAC ATA TCA ATA CAG GAC G	
MJK 274	OL_FlaB-B_R	ATT TGC TTC CTC TTC TAA GAA TCG AAT TAC AGC AGT GAT AAG GCC ACT TGA G	construction of in-frame insertion vectors pNPTS138-R6KT-double-*flaB*$_1$ - for Δ*flaB*$_1$ and pNPTS138-R6KT-double-*flaB*$_1$ - for Δ*flaAB*$_1$
MJK 275	OL_B-FlaB_F	TTC GAT TCT TAG AAG AGG AAG CAA ATA TGG CAA TTA CCG TGA ATA CAA ACG TTA C	
MJK 276	OL_B-HOM_R	GAT ATG TTC TGC TTA GTA AAT TAT AAA AGA CTC AAT GCC ACT TGA GGC	
MJK 277	check_inBsth_R	GCG ATC CTA CCT TCA ATG CAG	check primer for double flagellin constructs (*flaA*$_1$ and/or *flaB*$_1$)
MJK 278	check_outBsth_R	AGT AGG CAA AAG CAA CGT CCT G	
MJK 279	check_inAsth_R	CGT TGT TTG TGC CTA CGC TG	
FR 44	NheI_rpoN_KO_fw	GTA GCT AGC GAA GAA CGC GAA GAA GAA CTG G	construction of in-frame deletion vector
FR 45	OL_rpoN_KO_rv	GCT ATA AAC TCG CTT TCA TGT TAC CGC TGA TC	

FR 46	OL_rpoN_KO_fw	CAT GAA AGC GAG TTT ATA GCC TAC TAT GGA TAT CGG	pNPTS138-R6KT-*rpoN*-KO
FR 47	PspOMI_rpoN_KO_rv	TCC GGG CCC CGT TAC CTA TAC CTG TGC TCC	
FR 76	check_rpoN_fw	CTA TGC ATC TTC GCG CCC G	check primer for *rpoN*
FR 77	check_rpoN_rv	CGC TTT CAT GTT ACC GCT GAT C	
B 262	EcoRI-fliA1-KO-fwd	CGA ATT CCG TGC TTT CAG TGA GAT GCG	construction of in-frame deletion vector pNPTS138-R6KT-*fliA₁-KO*
B 263	OL-fliA1-KO-rev	GAG CTT AGC GGC TTT ATT CAC TCG TTT TTT CCT C	
B 264	OL-fliA1-KO-fwd	AAT AAA GCC GCT AAG CTC AAG CAC TGG ACA TAA	
B 265	PspOMI-fliA1-KO-rev	TCC GGG CCC CAA CTA AAT GCT GAG CCT GCT C	
B 266	Check-fliA1-KO-fwd	GCA CCC AAG GTA TGG TGG AAC	check primer for *fliA₁*
B 267	Check-fliA1-KO-rev	TGC AAG TTC TCT AAA TAA ATC ATC AGC	
FR 68	NheI_flrA1_KO_new_fw	GTA GCT AGC AGT AAT AGT TTG AAC ATG GAT GAA GG	construction of in-frame deletion vector pNPTS138-R6KT-*flrA*-KO
FR 69	OL_flrA1_new_rv	AAG ACT ATT CAT CTG TTT GCA TCA TTC AGT AGG C	
FR 70	OL_flrA1_new_fw	GCA AAC AGA TGA ATA GTC TTT TGC ATT TTT AGT TAT ATT ATT G	
FR 71	PspOMI_flrA1_KO_rv	TCC GGG CCC CAG ATA ACC GCT GCA GAT GTG	
FR 34	Check-flrA1-KO-fwd_new	GCT AAC TTG AAT AAT GAT GTG AAA GC	check primer for *flrA*
FR 35	Check-flrA1-KO-rev_new	CCG GAG TTA AAG GAG TAA TGG C	
FR 134	NheI-flrBC1-KO-fwd	GTA GCT AGC CGC ACT TGT ACC GTC GCT TC	construction of in-frame deletion vector pNPTS138-R6KT-*flrBC*-KO
FR 135	OL-flrBC1-KO-rev	CAC TAG CAA ACA GCT CGT ATG CGA GAT ATG GGG	
FR 136	OL-flrBC1-KO-fwd	TCG CAT ACG AGC TGT TTG CTA GTG TGC TTA AAT GTC	
FR 137	PspOMI-flrBC1-KO-rev	TCC GGG CCC GGA ACA TGC ATG GTC TGG CAA C	
FR 138	Check-flrBC1-KO-fwd	GCA GAT TGC CAA CGC AAG GAT C	check primer for *flrBC*
FR 139	Check-flrBC1-KO-rev	CTA CGT GTG TTG CAA GAG CGA G	
MK 98	EcoRV_KO_Maf1_US_fw	GAA TTC GTG GAT CCA GAT GTC GGA GGC CAG TTT AGT TG	construction of in-frame deletion vector pNPTS138-R6KT-*maf1*-KO
MK 99	OL_KO_Maf1_US_rv	AAA GTG GTG GGG TTT GTA GTT GCA TAT TTT CCT C	
MK 100	OL_KO_Maf1_DS_fw	ACT ACA AAC CCC ACC ACT TTA GCC CTA ATA TTG	
SH 555	Maf1 dwn rev	GCC AAG CTT CTC TGC AGG AT TTG CTA CGA ACA CCG GCA CC	
MK 101	chk_KO_Maf1_fw	CGA CAT TAC CAT AAC GAA CTG C	check primer

SH 557	Maf1 check dwn	TAA AGG TGC TGG CAC ATA GAG G	for *maf-1*
MK 94	EcoRV_KO_ PseG_US_fw	GAA TTC GTG GAT CCA GAT GAT ACT GAC GTT GCC TTT ATA TC	construction of in-frame deletion vector pNPTS138-R6KT-*pseG*-KO
MK 95	OL_KO_PseG_ US_rv	TTT AAC TTT CGG CCA ATT TCA TAA CTT TCC TTG	
MK 96	OL_KO_PseG_ DS_fw	GAA ATT GGC CGA AAG TTA AAT CAT GCA GGT AAT C	
MK 102	EcoRV_KO_ PseG_DS_rv	CAA GCT TCT CTG CAG GAT GAA AAC TCA CTA TCA ACA CCG C	
MK 97	chk_KO_PseG_ fw	CCA ACA CTA AAC CCG AGT TTC	check primer for *pseG*
SH 548	PseG check fw	TGT ACA TCC ATA ATG CAC TCG TCC	
MJK 145	XbaI-nC_ RBS_fliS_F	AAT GAA TAG TTC GAC AAA AAT AGG AGG GCA AAT ATG AGA GGA TCG CTG CAA TCA TAT CG	construction of overproduction vector pBTOK-RBS-*fliS*$_1$-RBS-*flaA*$_1$-T174C
MJK 146	OL_fliS_to_flaA 1_R	CAT ATT TGC CCT CCT TCC TAA ATT ACC TTA ACT CGC GTC GG	
MJK 147	OL_flaA1_to_ fliS_F	GGA AGG AGG GCA AAT ATG GCT ATT ACA GTT AAT ACC AAC GTG ACT TCG	
MJK 148	Bsp- nC_flaA1_R	GGA GTC CAA GCT CAG CTA ATG AAA TTA TCC CAG TAG TGA CAG CGC	
FR 116	NheI_fliS1_fw	GTA GCT AGC TGG GTC GCA AGC AAT TTT ATT GC	construction of in-frame deletion vectorpNPTS138-R6KT-fliS1-KO
FR 117	OL_fliS1_KO_rv	GAG AGG ATC GGC GAG TTA AGG TAA TTT AGG ACG	
FR 118	OL_fliS1_KO_fw	CTT AAC TCG CCG ATC CTC TCA TAA ATA CCT ACC	
FR 119	PspOMI_fliS1_ rv	TCC GGG CCC TTG CTG ACC AAA GGG AAG CC	
FR 140	check_fliS1_fw	GCT TAG TTT GAT TAG CAC TTG TAG G	check primer for *fliS*$_1$
FR 141	check_fliS1_rv	CTG AAG ATA TGT CCA GTA TTG AAG C	
MJK 141	EcoRV_fliS_C_ F	GAA TTC GTG GAT CCA GAT AGT ATC TTC AAG GAC AGC TTG ACC	construction of in-frame insertion vector pNPTS138-R6KT-*fliS*$_1$-FLAG
MJK 142	OL_-STP_ FLAG_fliS_R	AAT ATC ATG ATC TTT ATA ATC GCC ATC ATG ATC TTT ATA ATC ACT CGC GTC GGA ATG AGA GG	
MJK 143	OL_+STP_FLA G_fliS_F	ATT ATA AAG ATC ATG ATA TTG ATT ATA AAG ATG ATG ATG ATA AAT AAG GTA ATT TAG GAC GGG TCA AGG G	
MJK 144	EcoRV_fliS_KI_ R	CAA GCT TCT CTG CAG GAT CAA ACA ATA ATA TCG GTT GCC ACG C	
MJK 149	Check_fliS1- Flag-F	GTC AAC TTT TAC TCG ATG TGC TGG	check primer for *fliS*$_1$
MJK 150	Check_fliS1- Flag-R	TCT TCA ATT TGT CCA AGC ACA TTG G	
MJK 1	OL_flaA1_S129 N_N134T_rv	AGT AGT ACC AAA AGC GGT ATT GTC ACC AAT GG	construction of in-frame insertion vector

MJK 2	OL_flaA1_S129 N_N134T_fw	GAC AAT ACC GCT TTT GGT ACT ACT AAA CTG ATG AC	pNPTS138-R6KT-*flaA*1-S129N-N134T
MJK 3	OL_flaB1_N129 S_T134N_rv	AGT ATT ACC AAA GGC CGT ACT TGT ACC GAT TGC	construction of in-frame insertion vector
MJK 4	OL_flaB1_N129 S_T134N_fw	ACA AGT ACG GCC TTT GGT AAT ACT AAA TTA CTT GAT GG	pNPTS138-R6KT-*flaB*1-N129T-T134N
MJK 5	flaA1_qPCR_fw	GTT GGT ACC AAT AAT GCG GGG AG	qRT-PCR amplifying DNA fragments of *flaA*1 (Sputcn32_2586)
MJK 6	flaA1_qPCR_rv	CTA AAC GGT TTT GTT TAG CAC CTA ACG	
		Product length: 151 bp; efficiency: 1.98	
MJK 13	flaB1_neu_qPC R_fw	CTG CGA TTG ATG CTG CAA TTA AAA CC	qRT-PCR amplifying DNA fragments of *flaB*1 (Sputcn32_2585)
MJK 14	flaB1_neu_qPC R_rv	GCA ATA CTT GGT TCT TGG TCA TTT GC	
		Product length: 189 bp; efficiency: 2.00	
FR 233	gyrA_qPCR_fw	CAG AAT CGC CTG AGC TTG TTG C	qRT-PCR amplifying DNA fragments of *gyrA* (Sputcn32_2070)
FR 234	gyrA_qPCR_rv	GAG CAA GGT TGG GAA TTA GGC C	
		Product length: 144 bp; efficiency: 2.01	

Supplementary Table 4: Summary of screw formation, helix parameter and qPCR data and statistics

**Screw formation 0% Ficoll (Fig. 4)**				
Measurements				
Sample	Screw count	Regular count	Screw (%)	N
Wild type	12	287	4.01	299
FlaB-only	153	152	50.16	305
FlaAAB	5	292	1.68	297
Statistics*				
Combination	t	df	*P*-value	Test
Wild type vs. FlaB-only	NA	NA	2.32E-26	Fisher's exact test
Wild type vs. FlaAAB	NA	NA	0.139	Fisher's exact test
FlaB-only vs. FlaAAB	NA	NA	2.93E-32	Fisher's exact test

**Screw formation 15% Ficoll (Fig. 4)**				
Measurements				
Sample	Screw count	Regular count	Screw (%)	N
Wild type	150	147	50.51	297
FlaB-only	270	40	87.10	310
FlaAAB	78	243	24.30	321
Statistics*				
Combination	t	df	*P*-value	Test
Wild type vs. FlaB-only	NA	NA	3.09E-05	Fisher's exact test
Wild type vs. FlaAAB	NA	NA	4.43E-06	Fisher's exact test
FlaB-only vs. FlaAAB	NA	NA	9.74E-19	Fisher's exact test

Screw formation 0 vs. 15% Ficoll (Fig. 4)				
Statistics*				
Combination	t	df	*P*-value	Test
Wild type vs. Wild type	NA	NA	2.53E-26	Fisher's exact test
FlaB-only vs. FlaB-only	NA	NA	1.99E-05	Fisher's exact test
FlaAAB vs. FlaAAB	NA	NA	3.57E-15	Fisher's exact test

Helix parameters: Arc length (Supplementary Fig. 6)				
Measurements				
Sample	Average (μm)	Median (μm)	SD (μm)	N
Wild type	6.67	6.66	0.75	50
FlaAAB	6.71	6.82	0.66	50
FlaB-only	5.75	5.71	0.77	50
FlaA-only	6.45	6.35	1.68	50
FlaBA	5.71	5.52	1.29	50
FlaBBA	6.67	6.54	1.41	50
Statistics*				
Combination	t	df	*P*-value	Test
Wild type vs. FlaAAB	0.30	98.00	0.763	t-test
Wild type vs. FlaB-only	-5.96	98.00	3.95E-08	t-test
Wild type vs. FlaA-only	-0.82	67.81	0.417	Welch's t-test
Wild type vs. FlaBA	-4.48	78.56	2.49E-05	Welch's t-test
Wild type vs. FlaBBA	0.00	74.53	1.000	Welch's t-test
FlaAAB vs. FlaB-only	6.63	98.00	1.88E-09	t-test
FlaAAB vs. FlaA-only	1.00	63.68	0.321	Welch's t-test
FlaAAB vs. FlaBA	4.83	72.68	7.48E-06	Welch's t-test
FlaAAB vs. FlaBBA	0.19	69.21	0.848	Welch's t-test
FlaB-only vs. FlaA-only	2.66	68.85	0.010	Welch's t-test
FlaB-only vs. FlaBA	0.19	79.95	0.849	Welch's t-test
FlaB-only vs. FlaBBA	3.98	75.83	1.55E-04	Welch's t-test
FlaA-only vs. FlaBA	2.46	98.00	0.016	t-test
FlaA-only vs. FlaBBA	0.68	98.00	0.496	t-test
FlaBA vs. FlaBBA	3.50	98.00	0.001	t-test

Helix parameters: Axis length (Supplementary Fig. 6)				
Measurements				
Sample	Average (μm)	Median (μm)	SD (μm)	N
Wild type	4.71	4.68	0.43	50
FlaAAB	4.82	4.97	0.48	50
FlaB-only	3.96	3.90	0.51	50
FlaA-only	4.72	4.54	1.16	50
FlaBA	4.28	4.22	0.99	50
FlaBBA	4.80	4.72	1.05	50

Statistics*				
Combination	t	df	*P*-value	Test
Wild type vs. FlaAAB	1.12	98.00	0.265	t-test
Wild type vs. FlaB-only	-7.95	98.00	3.18E-12	t-test
Wild type vs. FlaA-only	0.00	62.32	0.997	Welch's t-test
Wild type vs. FlaBA	-2.82	66.83	0.006	Welch's t-test
Wild type vs. FlaBBA	0.55	64.97	0.584	Welch's t-test
FlaAAB vs. FlaB-only	8.59	98.00	1.35E-13	t-test
FlaAAB vs. FlaA-only	0.57	65.55	0.568	Welch's t-test
FlaAAB vs. FlaBA	3.43	70.97	0.001	Welch's t-test
FlaAAB vs. FlaBBA	0.09	68.75	0.932	Welch's t-test
FlaB-only vs. FlaA-only	4.19	67.07	8.38E-05	Welch's t-test
FlaB-only vs. FlaBA	-2.00	72.87	0.049	Welch's t-test
FlaB-only vs. FlaBBA	5.06	70.51	3.17E-06	Welch's t-test
FlaA-only vs. FlaBA	2.01	98.00	0.047	t-test
FlaA-only vs. FlaBBA	0.40	98.00	0.692	t-test
FlaBA vs. FlaBBA	2.54	98.00	0.013	t-test

Helix parameters: Diameter (Supplementary Fig. 6)

Measurements				
Sample	Average (µm)	Median (µm)	SD (µm)	N
Wild type	0.57	0.58	0.05	50
FlaAAB	0.51	0.51	0.05	50
FlaB-only	0.60	0.60	0.05	50
FlaA-only	0.35	0.35	0.03	50
FlaBA	0.33	0.33	0.03	50
FlaBBA	0.37	0.37	0.03	50

Statistics*				
Combination	t	df	*P*-value	Test
Wild type vs. FlaAAB	-6.11	98.00	2.04E-08	t-test
Wild type vs. FlaB-only	2.44	98.00	0.017	t-test
Wild type vs. FlaA-only	-25.42	83.81	3.78E-41	Welch's t-test
Wild type vs. FlaBA	-28.18	76.30	4.45E-42	Welch's t-test
Wild type vs. FlaBBA	-22.60	83.98	1.86E-37	Welch's t-test
FlaAAB vs. FlaB-only	-8.86	98.00	3.55E-14	t-test
FlaAAB vs. FlaA-only	20.57	90.75	6.13E-36	Welch's t-test
FlaAAB vs. FlaBA	23.53	83.29	1.49E-38	Welch's t-test
FlaAAB vs. FlaBBA	17.47	90.90	8.27E-31	Welch's t-test
FlaB-only vs. FlaA-only	-29.05	85.77	3.65E-46	Welch's t-test
FlaB-only vs. FlaBA	32.10	78.15	9.18E-47	Welch's t-test
FlaB-only vs. FlaBBA	-26.16	85.94	1.08E-42	Welch's t-test
FlaA-only vs. FlaBA	2.27	98.00	0.025	t-test
FlaA-only vs. FlaBBA	3.63	98.00	4.52E-04	t-test
FlaBA vs. FlaBBA	6.17	98.00	1.55E-08	t-test

**Helix parameters: Pitch (Supplementary Fig. 6)**				
Measurements				
Sample	Average (µm)	Median (µm)	SD (µm)	N
Wild type	1.75	1.73	0.10	50
FlaAAB	1.66	1.67	0.09	50
FlaB-only	1.80	1.79	0.07	50
FlaA-only	1.18	1.18	0.04	50
FlaBA	1.18	1.17	0.05	50
FlaBBA	1.21	1.21	0.05	50
Statistics*				
Combination	t	df	*P*-value	Test
Wild type vs. FlaAAB	-4.31	98.00	3.91E-05	t-test
Wild type vs. FlaB-only	2.87	88.35	0.005	Welch's t-test
Wild type vs. FlaA-only	-37.47	65.98	3.42E-46	Welch's t-test
Wild type vs. FlaBA	-35.66	75.81	3.96E-49	Welch's t-test
Wild type vs. FlaBBA	-33.57	76.65	1.38E-47	Welch's t-test
FlaAAB vs. FlaB-only	-8.08	98.00	1.71E-12	t-test
FlaAAB vs. FlaA-only	34.48	68.92	3.37E-45	Welch's t-test
FlaAAB vs. FlaBA	32.58	79.83	7.16E-48	Welch's t-test
FlaAAB vs. FlaBBA	30.35	80.72	6.59E-46	Welch's t-test
FlaB-only vs. FlaA-only	-53.53	79.93	2.27E-64	Welch's t-test
FlaB-only vs. FlaBA	49.33	98.00	5.25E-71	t-test
FlaB-only vs. FlaBBA	-46.52	98.00	1.29E-68	t-test
FlaA-only vs. FlaBA	-0.08	98.00	0.939	t-test
FlaA-only vs. FlaBBA	3.26	98.00	0.002	t-test
FlaBA vs. FlaBBA	2.85	98.00	0.005	t-test

**Helix parameters: flagellar stub length (Supplementary Fig. 5)**				
Measurements				
Sample	Average (µm)	Median (µm)	SD (µm)	N
FlaA	0.94	0.91	0.29	50
FlaAA	1.53	1.53	0.43	50
FlaB	0.39	0.39	0.08	50
FlaBB	0.54	0.51	0.13	50
Statistics*				
Combination	t	df	*P*-value	Test
FlaA vs. FlaAA	7.95	85.57	7.01E-12	Welch's t-test
FlaA vs. FlaB	12.72	56.76	2.92E-18	Welch's t-test
FlaA vs. FlaBB	-8.75	68.38	9.08E-13	Welch's t-test
FlaAA vs. FlaB	18.09	52.50	3.26E-24	Welch's t-test
FlaAA vs. FlaBB	15.30	57.98	5.04E-22	Welch's t-test
FlaB vs. FlaBB	6.75	81.97	1.98E-09	Welch's t-test

qRT-PCR: Relative transcription level (RTL) flagellins (Supplementary Fig. 7)				
Measurements				
Sample	Average (RTL)	Median (RTL)	SD (RTL)	N
flaA	1.07	1.04	0.12	3
flaB	1.87	1.96	0.25	3
Statistics*				
Combination	t	df	*P*-value	Test
flaA vs. *flaB*	-4.12	4.00	0.015	t-test

* The color code indicates significant differences: blue shading = significant; yellow shading = not significant (Bonferroni corrected; *P*-value threshold = 0.05 divided by the number of tests).

Supplementary Table 5: Sequences of the synthetic flaA and flaB genes

**Synthetic gene**	*flaA*$_{\text{ECopt}}$
**Native gene**	*flaA* (Sputcn32_2586)
**Modifications**	The sequence was codon-optimized for *Escherichia coli* K-12 to prevent recombination with the native flagellin gene(s).<br>Threonine 174 was substituted by cysteine for fluorescent labeling.
**Sequence synthetic gene (5'-3')**	ATG GCT ATT ACG GTT AAC ACG AAT GTA ACA TCT ATG AAA GCT CAA<br>AAG AAC CTT AAC ACT AGC TCA AGC GGG CTG GCC ACT TCC ATG GAG<br>CGT TTA TCC AGT GGC CTG CGC ATT AAC AGT GCC AAA GAC GAC GCA<br>GCT GGC TTG GCT ATC TCC AAT CGC CTG AAC TCA CAA GTG CGC GGG<br>TTG GAT GTG GGC ATG CGC AAC GCT AAT GAC GCA ATC TCT ATC GCG<br>CAA ATC GCG GAA GGT GCC ATG CAG GAG CAA ACC AAC ATG CTG CAA<br>CGC ATG CGT GAT CTG ACC GTG CAG GCG GAA AAT GGT GCG AAC TCG<br>ACC GAT GAC CTG GAC GCA ATC CAA AAG GAA ATC GAT CAG TTA GCT<br>GAA GAG ATC ACA GCC ATT GGC GAT AGT ACC GCA TTC GGC AAT ACT<br>AAA CTT ATG ACT GGG AAT TTT TCT GCC GGC AAG ACC TTT CAA GTT<br>GGG CAC CAA GAA GGT GAA GAC ATT ACC ATC AGC GTA GGC ACA AAC<br>AAC GCC GGC AGC TTA ATG GTC AGT TGT CTT ACA ATC GCC ACA AGT<br>GGC GGG CGT TCG ACC GCA TTG GCG GCC ATC GAT GCT GCA ATC AAG<br>AAC ATT GAC AAC CAA CGC GCT GCT TTA GGA GCC AAA CAG AAC CGT<br>CTG GCG TAT AAC ATC AGC AAC TCC GCA AAT ACG CAG GCA AAC GTA<br>GCT GAC GCA AAG TCC CGT ATC GTG GAT GTT GAT TTT GCG AAG GAG<br>ACC TCA GTA ATG ACG AAA AAT CAG GTT TTG CAA CAG ACC GGA AGT<br>GCT ATG TTA GCC CAA GCC AAT CAG TTA CCC CAG GTG GCG CTG TCG<br>CTT CTG GGC TAA
**Sequence native gene (5'-3')**	ATG GCT ATT ACA GTT AAT ACC AAC GTG ACT TCG ATG AAG GCA CAG<br>AAA AAT TTA AAT ACG TCT AGT AGT GGT TTA GCA ACC TCT ATG GAA<br>CGT TTA TCA AGT GGC CTG CGC ATC AAT AGC GCC AAA GAC GAC GCC<br>GCT GGT TTA GCC ATT TCA AAT CGT CTA AAC AGT CAG GTA CGT GGT<br>TTA GAT GTG GGA ATG CGC AAT GCT AAT GAT GCG ATC TCC ATT GCC<br>CAG ATC GCT GAA GGT GCA ATG CAA GAG CAG ACT AAC ATG CTG CAA<br>CGT ATG CGT GAT TTG ACT GTA CAA GCT GAA AAC GGT GCA AAT AGC<br>ACC GAT GAC TTA GAT GCA ATA CAA AAA GAG ATC GAT CAA TTA GCT<br>GAA GAG ATT ACT GCC ATT GGT GAC AGT ACC GCT TTT GGT AAT ACT<br>AAA CTG ATG ACA GGG AAT TTT TCT GCG GGA AAA ACC TTC CAA GTA<br>GGG CAC CAA GAA GGT GAA GAT ATC ACT ATT TCC GTT GGT ACC AAT<br>AAT GCG GGG AGT TTA ATG GTT AGT ACA TTA ACG ATT GCA ACT TCA<br>GGT GGT CGC TCT ACG GCC CTC GCA GCC ATT GAT GCG GCA ATT AAA<br>AAT ATT GAT AAC CAA CGT GCA GCG TTA GGT GCT AAA CAA AAC CGT

	AAT ATT GAT AAC CAA CGT GCA GCG TTA GGT GCT AAA CAA AAC CGT<br>TTA GCC TAT AAC ATC AGT AAC AGT GCT AAC ACT CAA GCA AAC GTT<br>GCC GAT GCT AAG AGC CGT ATT GTC GAT GTC GAT TTT GCT AAA GAA<br>ACA TCA GTA ATG ACG AAA AAC CAA GTA TTA CAA CAA ACG GGT TCT<br>GCA ATG TTA GCG CAG GCT AAC CAA TTG CCT CAA GTT GCG CTG TCA<br>CTA CTG GGA TAA
**Synthetic gene**	*flaB*Ecopt
**Native gene**	*flaB* (Sputcn32_2585)
**Modifications**	The sequence was codon-optimized for *Escherichia coli* K-12 to prevent recombination with the native flagellin gene(s).<br>Threonine 166 was substituted by cysteine for fluorescent labeling.
**Sequence synthetic gene (5'-3')**	ATG GCA ATT ACC GTG AAT ACA AAC GTT ACC TCC CTT AAG GCC CAA<br>AAG AAC TTG AAC ACT TCG GCC AGC GGG TTG GCC ACA TCA ATG GAA<br>CGC CTG TCT TCT GGC CTT CGT ATT AAC GGC GCT AAG GAT GAC GCC<br>GCA GGG TTA GCA ATC TCC AAC CGC TTG AAT TCG CAA GTG CGC GGA<br>CTG GAC GTC GGC ATG CGT AAC GCG AAC GAC GCG ATT TCA ATT GCA<br>CAA ATT TCA GAA GGC GCA ATG CAA GAG CAA ACA AAT ATG CTG CAA<br>CGC ATG CGT GAC CTG ACG GTA CAG GCC GAG AAT GGG GCG AAT AGT<br>TCA GAC GAT CTT ACC TCC ATT CAA AAA GAA ATT GAC CAA TTA GCG<br>CTG GAG ATC ACG GCC ATC GGG ACA AAC ACC GCA TTT GGG ACA ACT<br>AAG TTG CTT GAT GGC ACC TTT TCG GCG GGC AAA ACT TTT CAG GTC<br>GGA CAT CAA TCG GGT GAG GAC ATC ACG ATC TCT GTA TCG AAG ACG<br>TGT GCG TCT GCA TTG AAG GTA GGA TCG CTG GAC ATC AAG GGT TCG<br>GCT CGC GCC TCA GCC TTG GCA GCA ATC GAC GCT GCT ATC AAA ACG<br>ATT GAT AGT CAA CGC GCT GAC TTG GGG GCT AAG CAA AAC CGC TTG<br>GCG TAC AAC ATT TCC AAC TCA GCT AAC ACA CAG GCA AAC ATC TCT<br>GAC GCC AAA TCC CGT ATC GTA GAT GTA GAT TTT GCT AAG GAA ACT<br>AGC CAG ATG ACA AAG AAT CAG GTT TTG CAG CAA ACG GGT TCC GCC<br>ATG TTG GCA CAG GCG AAT CAG TTG CCT CAA GTG GCA TTG AGT CTT<br>TTA TAA
**Sequence native gene (5'-3')**	ATG GCC ATT ACA GTA AAC ACT AAC GTA ACA TCT TTA AAA GCA CAG<br>AAA AAC CTA AAT ACT TCA GCG AGC GGT TTG GCC ACT TCC ATG GAA<br>CGT TTA TCC AGT GGT CTG CGT ATT AAC GGT GCA AAG GAC GAT GCG<br>GCA GGT TTA GCA ATT TCT AAC CGC TTA AAT AGC CAA GTC CGT GGC<br>TTA GAT GTG GGT ATG CGT AAC GCT AAC GAT GCT ATC TCT ATC GCC<br>CAA ATT TCT GAA GGT GCG ATG CAA GAA CAA ACT AAC ATG CTG CAA<br>CGT ATG CGT GAC TTA ACC GTC CAA GCA GAA AAC GGT GCT AAT AGT<br>TCA GAT GAC TTA ACG TCA ATA CAA AAA GAG ATC GAT CAG TTA GCA<br>TTA GAA ATC ACA GCA ATC GGT ACA AAT ACG GCC TTT GGT ACT ACT<br>AAA TTA CTT GAT GGC ACT TTC TCT GCT GGT AAG ACT TTC CAA GTA<br>GGG CAC CAA TCA GGT GAA GAT ATT ACG ATT TCT GTG TCA AAA ACC<br>ACT GCA TCA GCA TTA AAA GTT GGT AGT TTA GAT ATT AAA GGC TCT<br>GCT CGA GCC TCT GCA CTG GCT GCG ATT GAT GCT GCA ATT AAA ACC<br>ATT GAT AGT CAG CGT GCG GAT CTA GGT GCT AAG CAA AAC CGC TTA<br>GCC TAT AAC ATC AGT AAT AGT GCT AAC ACT CAG GCC AAC ATT TCT<br>GAT GCT AAG AGT CGT ATT GTG GAT GTG GAT TTT GCG AAA GAA ACA<br>TCG CAA ATG ACC AAG AAC CAA GTA TTG CAA CAA ACG GGT TCT GCT<br>ATG TTA GCC CAA GCT AAC CAA TTA CCT CAA GTG GCC TTA TCA CTG<br>CTG TAA

Supplementary Table 6: Parameters used for the simulations

Flagellin type		FlaB		FlaA
Parameter	Description	Stretched	Coiled	Stretched
R	Helical radius	0.315 μm	0.42 μm	0.175 μm
P	Helical pitch	1.91 μm	1.43 μm	1.18 μm
L_c	Contour length	6.5 μm		
A	Bending rigidity	3.5 pN μm^2		
C	Twisting rigidity	3.5 pN μm^2		
K	Stretching stiffness	10000 pN μm^{-1}		
$\gamma_\perp$	Friction coefficient, normal to flagellum	2.85 η	2.80 η	3.25 η
$\gamma_\parallel$	Friction coefficient, parallel to flagellum	1.61 η	1.57 η	1.86 η
γ_r	Rotational friction coefficient	0.0012 η		
η	Viscosity	1 mPa s (water at 20 °C)		
h_0	Segment length	0.125 μm		

Supplementary References

1. Sun, L. *et al.* Two residues predominantly dictate functional difference in motility between *Shewanella oneidensis* flagellins FlaA and FlaB. *J. Biol. Chem.* **289**, 14547-14559 (2014).
2. Miller, V. L. & Mekalanos, J. J. A novel suicide vector and its use in construction of insertion mutations: Osmoregulation of outer membrane proteins and virulence determinants in *Vibrio cholerae* requires toxR. *J. Bacteriol.* **170,** 2575–2583 (1988).
3. W. Metcalf, University of Illinois, Urbana-Champaign
4. Fredrickson, J. K. *et al.* Biogenic iron mineralization accompanying the dissimilatory reduction of hydrous ferric oxide by a groundwater bacterium. *Geochim. Cosmochim. Acta.* **62,** 3239–3257 (1998).
5. Bubendorfer, S., Koltai, M., Rossmann, F., Sourjik, V. & Thormann, K. M. Secondary bacterial flagellar system improves bacterial spreading by increasing the directional persistence of swimming. *Proc. Natl. Acad. Sci. USA* **111,** 11485–11490 (2014).
6. Kühn, M. J., Schmidt, F. K., Eckhardt, B. & Thormann, K. M. Bacteria exploit a polymorphic instability of the flagellar filament to escape from traps. *Proc. Natl. Acad. Sci. USA* **114,** 6340–6345 (2017).
7. Venkateswaran, K. *et al.* Polyphasic taxonomy of the genus *Shewanella* and description of *Shewanella oneidensis* sp. nov. *Int. J. Syst. Evol. Microbiol.* **49,** 705–724 (1999).
8. Bubendorfer, S. *et al.* Analyzing the modification of the *Shewanella oneidensis* MR-1 flagellar filament. *PLoS ONE* **8**, e73444 (2013).
9. Lassak, J., Henche, A. L., Binnenkade, L. & Thormann, K. M. ArcS, the cognate sensor kinase in an atypical arc system of *Shewanella oneidensis* MR-1. *Appl. Environ. Microbiol.* **76,** 3263–3274 (2010).
10. Rossmann, F. *et al.* The role of FlhF and HubP as polar landmark proteins in *Shewanella putrefaciens* CN-32. *Mol. Microbiol.* **98,** 727–742 (2015).
