# Supplementary material for: Spatial arrangement of several flagellins within bacterial flagella improves motility in different environments
Source: Nat Commun. 2018 Dec 18;9:5369. doi: 10.1038/s41467-018-07802-w (PMC6299084; doi:10.1038/s41467-018-07802-w)
Supplement: Supplementary file 3 — Description of Additional Supplementary Files [file 41467_2018_7802_MOESM3_ESM.pdf]

Description of Additional Supplementary Files

File Name: Supplementary Movie 1
Description: Corresponding movie to Fig. 3 panels i and j of the main manuscript. The movie shows stereotypical cell tracks for wild-type cells in medium with increased viscosity (left) and regular medium (right). Swimming speed is encoded by color according to the color bar on the right.

File Name: Supplementary Movie 2
Description: Movie extracted from the numerical simulation. FlaA-only (left) and FlaBonly flagellar filaments (right) rotate clockwise with a motor torque of 6.5 pNµm (see also Fig. 4 of the main manuscript). The only differences between the two simulations are the helix parameters that were experimentally measured (Supplementary Table 6). The simulation is visualized using OVITO.
