# Supplementary material for: Spatial arrangement of several flagellins within bacterial flagella improves motility in different environments
Source: Nat Commun. 2018 Dec 18;9:5369. doi: 10.1038/s41467-018-07802-w (PMC6299084; doi:10.1038/s41467-018-07802-w)
Supplement: Supplementary file 6 — Reporting Summary [file 41467_2018_7802_MOESM6_ESM.pdf]

natureresearch

Corresponding author(s): Kai M Thormann
Bruno Eckhardt
Laurence G Wilson

Reporting Summary

Nature Research wishes to improve the reproducibility of the work that we publish. This form provides structure for consistency and transparency in reporting. For further information on Nature Research policies, see Authors & Referees and the Editorial Policy Checklist.

Statistical parameters

When statistical analyses are reported, confirm that the following items are present in the relevant location (e.g. figure legend, table legend, main text, or Methods section).

n/a	Confirmed	
☐	☒	The exact sample size (n) for each experimental group/condition, given as a discrete number and unit of measurement
☐	☒	An indication of whether measurements were taken from distinct samples or whether the same sample was measured repeatedly
☐	☒	The statistical test(s) used AND whether they are one- or two-sided<br>*Only common tests should be described solely by name; describe more complex techniques in the Methods section.*
☒	☐	A description of all covariates tested
☐	☒	A description of any assumptions or corrections, such as tests of normality and adjustment for multiple comparisons
☐	☒	A full description of the statistics including central tendency (e.g. means) or other basic estimates (e.g. regression coefficient) AND variation (e.g. standard deviation) or associated estimates of uncertainty (e.g. confidence intervals)
☐	☒	For null hypothesis testing, the test statistic (e.g. F, t, r) with confidence intervals, effect sizes, degrees of freedom and P value noted<br>*Give P values as exact values whenever suitable.*
☒	☐	For Bayesian analysis, information on the choice of priors and Markov chain Monte Carlo settings
☒	☐	For hierarchical and complex designs, identification of the appropriate level for tests and full reporting of outcomes
☒	☐	Estimates of effect sizes (e.g. Cohen's d, Pearson's r), indicating how they were calculated
☐	☒	Clearly defined error bars<br>*State explicitly what error bars represent (e.g. SD, SE, CI)*

Our web collection on statistics for biologists may be useful.

Software and code

Policy information about availability of computer code

Data collection	QRT-PCR data was collected using Real-Time CFX Manager 2.1. Flagellar parameters and screw formation measurements were done manually using the ImageJ distribution Fiji (Version 1.51). Microscopy images were acquired using VisiView (Version 4.1.0.6).<br>All digital holography software (code to prouce three-dimensional tracks from raw videos) is documented in previous publications (https://doi.org/10.1364/OE.20.016735, https://doi.org/10.1364/OE.25.028489).<br>Simulation is described in detail in a previous publication (https://doi.org/10.1073/pnas.1701644114)
Data analysis	Statistic parameter calculations for the experimental measurements were carried out in Excel 2013 and R (Version 3.3.2), as stated in the Methods section.<br>For tracking, no customised data analysis routines were used, and all remaining parameters are specified in the supplementary materials of the original submission.<br>Simulation code is available upon request, as stated in the manuscript.

For manuscripts utilizing custom algorithms or software that are central to the research but not yet described in published literature, software must be made available to editors/reviewers upon request. We strongly encourage code deposition in a community repository (e.g. GitHub). See the Nature Research guidelines for submitting code & software for further information.

Data

Policy information about availability of data

All manuscripts must include a data availability statement. This statement should provide the following information, where applicable:

- Accession codes, unique identifiers, or web links for publicly available datasets
- A list of figures that have associated raw data
- A description of any restrictions on data availability

All original data underlying Fig. 1, 3 and 4, Supplementary Figures 2-13 and Supplementary Movies 1 & 2 will be made available upon request. The holographic analysis datasets produced during the current study are available in the University of York repository, https://doi.org/10.15124/675b6083-2a6e-43d1-8511-b7c18bb1be9a.

Field-specific reporting

Please select the best fit for your research. If you are not sure, read the appropriate sections before making your selection.

☒ Life sciences ☐ Behavioural & social sciences ☐ Ecological, evolutionary & environmental sciences

For a reference copy of the document with all sections, see nature.com/authors/policies/ReportingSummary-flat.pdf

Life sciences study design

All studies must disclose on these points even when the disclosure is negative.

Sample size	No statistical methods were used to predetermine the sample size. Instead, sample sizes were estimated based on published work with similar measurements. Flagella measurement sample sizes varied drastically among publications. At the sample sizes chosen in our manuscript, we found that measuring more samples did not change the mean values significantly.
Data exclusions	Single flagellar filaments were excluded from the final data set of filament parameters if the total filament length was shorter than at least one complete helix turn. For measuring the proportion of the proximal filament segment, all filaments shorter than 4 µm were excluded. For the additional measurements of the newly generated mutants (for the revised manuscript) wild-type samples were measured again as a control. To adjust sample sizes of the wild-type data sets, the data was combined, randomised and reduced accordingly. Mean values did not change significantly by this procedure.
Replication	Generally, experiments were replicated at least three times in independent experiments to verify the reproducibility of the findings. All attempts at replication were successful. The parameters of the wild-type flagellar filaments were found to be highly similar among replicates.
Randomization	Filament parameter and screw formation samples were chosen randomly from the acquired images or image sequences. Experimental groups were the different mutant strains, so they were not actively allocated furthermore.
Blinding	Blinding was not relevant as samples were not allocated actively into groups other than the obvious genetic differences of the mutant strains.

Reporting for specific materials, systems and methods

Materials & experimental systems

n/a	Involved in the study	
☒	☐	Unique biological materials
☐	☒	Antibodies
☒	☐	Eukaryotic cell lines
☒	☐	Palaeontology
☒	☐	Animals and other organisms
☒	☐	Human research participants

Methods

n/a	Involved in the study	
☒	☐	ChIP-seq
☒	☐	Flow cytometry
☒	☐	MRI-based neuroimaging

Antibodies

Antibodies used	Custom polyclonal rabbit anti-flagellin: Eutogentec, ZDE # 10072, Rabbit # 366, SAB 23.06.2010<br>Goat anti-rabbit IgG (H+L) Secondary Antibody HRP conjugate: Invitrogen, Prod # 31460, Lot # RJ242536<br>Monoclonal mouse anti-FLAG(R) M2-Peroxidase (HRP) antibody: Sigma Aldrich, Prod # A8592, Clone # M2, Lot # SLBS6330

Validation

Anti-flagellin: The specificity of the anti-flagellin antibody was confirmed using wild-type and flagellin (A and B) deletion samples. This antibody has already been used in the following publications: https://doi.org/10.1371/journal.pone.0073444; https://doi.org/10.1073/pnas.1701644114.
Anti-FLAG and Goat anti-rabbit antibodies: See product information and specifications sheet on the manufacturer's website.
